# Proteomic Profiling of Extracellular Vesicles Separated from Plasma of Former National Football League Players at Risk for Chronic Traumatic Encephalopathy

**DOI:** 10.14336/AD.2020.0908

**Published:** 2021-09-01

**Authors:** Satoshi Muraoka, Annina M DeLeo, Zijian Yang, Harutsugu Tatebe, Kayo Yukawa-Takamatsu, Seiko Ikezu, Takahiko Tokuda, David Issadore, Robert A Stern, Tsuneya Ikezu

**Affiliations:** ^1^Department of Pharmacology and Experimental Therapeutics, Boston University School of Medicine, Boston, MA, USA.; ^2^Deprtment of Bioengineering, University of Pennsylvania, Philadelphia, PA, USA.; ^3^Department of Functional Brain Imaging Research, National Institute of Radiological Sciences, National Institutes for Quantum and Radiological Science and Technology, Chiba, JAPAN.; ^4^Department of Neurology, Boston University Alzheimer’s Disease and CTE Centers, Boston University School of Medicine, Boston, MA, USA.; ^5^Departments of Anatomy & Neurobiology and Neurosurgery, Boston University School of Medicine, Boston, MA, USA.; ^6^Center for Systems Neuroscience, Boston University, Boston, MA, USA.

**Keywords:** chronic traumatic encephalopathy, extracellular vesicles, machine learning, plasma, proteome

## Abstract

Chronic Traumatic Encephalopathy (CTE) is a tauopathy that affects individuals with a history of exposure to repetitive head impacts, including National Football League (NFL) players. Extracellular vesicles (EVs) are known to carry tau in Alzheimer’s disease and other tauopathies. We examined protein profiles of EVs separated from the plasma of former NFL players at risk for CTE. EVs were separated from the plasma from former NFL players and age-matched controls using size-exclusion chromatography. Label-free quantitative proteomic analysis identified 675 proteins in plasma EVs, and 17 proteins were significantly differentially expressed between former NFL players and controls. Total tau (t-tau) and tau phosphorylated at threonie181 (p-tau_181_) in plasma-derived EVs were measured by ultrasensitive immunoassay. Level of t-tau and p-tau_181_ in EVs were significantly different, and the area under the receiver operating characteristic curve (AUC) of t-tau and p-tau_181_ showed 0.736 and 0.715, respectively. Machine learning analysis indicated that a combination of collagen type VI alpha 3 and 1 chain (COL6A3 and COL6A1) and reelin (RELN) can distinguish former NFL players from controls with 85% accuracy (AUC = 0.85). Based on the plasma EV proteomics, these data provide protein profiling of plasma EVs for CTE, and indicate combination of COL6A3, RELN and COL6A1 in plasma EVs may serve as the potential diagnostic biomarkers for CTE.

Chronic Traumatic Encephalopathy (CTE) is a neurodegenerative tauopathy that is associated with exposure to repetitive head impacts such as those sustained by contact or collision sport athletes, including boxers and American tackle football, soccer, rugby, and ice hockey players [1]. CTE is characterized by neurofibrillary tangles (NFTs) composed of perivascular neuronal or astrocytic deposition of phosphorylated microtubule-associated protein tau (p-tau). In later stages, the p-tau depositions become widespread and lead to neuronal loss and neurodegeneration [1-3]. Recently, it has been reported that the conformation of tau filaments in CTE is distinct from other tauopathies such as Alzheimer’s disease (AD) [4-6]. The β-helix of tau filaments in CTE have differently conformation from tau filaments of Alzheimer’s disease and creates a hydrophobic cavity. The differently cavity from Alzheimer’s disease may contribute the progression of CTE [4]. At this time, CTE can only be diagnosed by neuropathologic examination. However, a recent study provided preliminary support for the use of the positron emission tomography (PET) p-tau ligand flortaucipir to detect CTE in living former national football league (NFL) players [7]. PET imaging for routine diagnostic or screening purposes is, however, limited due to its expense and lack of availability. On the other hand, accessible fluid biomarkers could be more useful for the detection and diagnosis of CTE in those at presumed risk through prior repetitive head impact exposure. Several fluid biomarkers, including the measurement of total tau (t-tau), p-tau and β-amyloid (Aβ) in cerebrospinal fluid (CSF) or plasma, have shown promise in the diagnosis and early detection of AD [8-11]. In initial studies of CSF and plasma levels of tau in the detection of CTE, however, there were no significant differences found between former NFL players and controls in t-tau and p-tau levels [11,12].

Extracellular vesicles (EV), including exosomes (50-150nm), ectosomes/microvesicles (150-1000nm) and apoptotic bodies (1000-5000nm) are released into the extracellular space by almost all cell types in the central nervous system (CNS), including neurons and glia [13-15]. These vesicles are found in saliva, Urine, blood and CSF [16-19]. Such vesicles contain many types of nucleic acids, lipids, and proteins that can be transferred to recipient cells as a form of cell-to-cell communication [20,21]. In the CNS, it has been reported that AD-associated pathogenic proteins, including tau and Aβ oligomers, are present in brain EVs and play important roles in AD pathogenesis [22-24]. Models of neuron-to-neuron transfer of tau seeds by EVs have been reported in AD [23,25]. A recent study reported that the Bridging Integrator 1 (BIN1) protein, which is associated with the progression of tau pathology, may alter tau clearance by promoting the release of tau-enriched microglial EVs [26].

Although morphological, proteomic, or RNA analyses were previously performed with EVs extracted from plasma or serum samples from patients with neurodegenerative diseases [27-29], no quantitative proteomics database have yet been established for former NFL players at risk for CTE. Herein, we provide the first proteomic profiling of EVs separated from former NFL players’ plasma samples.

## MATERIALS AND METHODS

### Sample selection, blood sampling, and clinical measures

The plasma samples were obtained from the National Institutes of Health-funded study, “Diagnosing and Evaluating Traumatic Encephalopathy using Clinical Tests” (DETECT) at Boston University School of Medicine. Participants included in the current study were 30 former NFL players with cognitive and neuropsychiatric symptoms, and a control group of 25 asymptomatic age-matched men without a history of contact/collision sports or traumatic brain history. The DETECT study procedures have been described elsewhere [10-12]. The selection of participants for the current study from the larger DETECT Study cohort was based on availability of an adequate number of plasma aliquots for EV separation remaining in the study freezer at the time of this study. At the time of participation, non-fasting blood samples were collected into plastic dipotassium EDTA tubes and processed according to standard procedures. Plasma was aliquoted and frozen at -80ºC [30]. All participants were administered a neuropsychological test battery that assessed the major cognitive domains (i.e., attention, executive function, verbal and visual episodic memory, language, visuospatial function) and standardized interview-based and self-report measures of mood and behavior. Neuropsychological test raw scores were transformed to standard scores corrected for age, gender, and/or education using published normative data. Principal component analysis was performed to generate four clinical factor composite scores: Behavioral/Mood, Psychomotor Speed/Executive Function, Verbal Memory, and Visual Memory. These clinical factor scores were included in the present study [31]. A list of the tests and details of the factor score generation are described in Alosco *et al* [32]. The Institutional Review Board at Boston University Medical Campus approved the protocol and all participants provided informed consent (Institutional Review Board (IRB); H-32363).

### Separation of EVs from human plasma samples

The EV fraction was separated from the plasma samples using qEV original columns (#qEVoriginal-5 Pack IZON Science, MA, USA). Briefly, 500μL of plasma was centrifuged at 1,500× *g* for 10 min at 4°C, then the supernatant was centrifuged at 10,000× *g* for 20 min at 4°C. The EV fraction was separated from the supernatant using the qEV columns and then eluted with 500μL of double-filtered PBS (dfPBS, with 0.22-μm pore-size [#SLGP033RS EMD Millipore, USA]). Particles and protein concentrations were determined by nanoparticle tracking analysis and bicinchoninic acid assay, respectively.

### Nanoparticle Tracking Analysis (NTA)

All samples were diluted in dfPBS at least 1:10 to obtain particles within the target reading range of Nanosight 300 (Malvern Panalytical Inc., UK) and sCMOS camera:10-100 particles per frame. Using a stage pump system, four 30-second videos were taken for each sample at 21°C and set screen gain to 1.0 and adjust the camera level unit the particle in the screen can be clearly seen. Analysis of particle counts was carried out in the Nanosight NTA 3.2 software (Malvern Panalytical Inc., UK) with a detection threshold of 5.

### Transmission electron microscopy (TEM)

The EV separated from former NFL player and control plasma were analyzed by TEM. EV fractions were subjected to electron microscopy as described [33]. Briefly, 5μL of EV samples were adsorbed for 1 minute to a carbon-coated grid that has been made hydrophilic by a 30-s exposure to a glow discharge. Excess liquid was removed with a filter paper (Whatman no. 1), and samples were stained with 0.75 % uranyl formate for 30 s. After excess uranyl formate was removed with a filter paper, grids were examined in a TecnaiG2 Spirit BioTWIN (FEI), and images were recorded with an AMT 2k CCD camera. (AMT, Woburn, MA).

### Assessment of protein concentration

The bicinchoninic acid assay (#23225 Thermo Fisher Scientific, USA) was used to determine protein concentration for each sample. EVs were lysed with TET buffer (50mM Tris-HCl pH 7.5, 2mM EDTA, 1% Triton X-100) for 15 min on ice before loading into the assay. All assays were allowed to incubate at 60°C for 30 min before protein concentration was read in a Biotek SynergyMix at 562 nm.

## MASS SPECTROMETRY

### SDS-PAGE and in-gel digestion

Proteomics analysis used the high purity EV fraction which was identified by NTA and protein concentration. Ice-cold 100% (w/v) acetone (#179124 Sigma-Aldrich, USA) was added to the high purity EV fraction (qEV fraction #8 or #9) to a final concentration of 20% of acetone, then the mixed sample was incubated for 16hrs at -20°C and was centrifuged at 20,000× *g* for 15 min at 4°C. The pellet was then washed once with 80% ice-cold acetone. After drying, the pellet was resuspended in Laemmli sample buffer (#1610747 Bio-Rad, USA). Subsequently, the samples were run in a 10% gel (#456-1033 Bio-Rad, USA) until the dye front was 10 mm from the top of the gel. The gels were washed twice with distilled water, fixed with fixation buffer (50% methanol, 10% acetic acid and 40% H_2_O), and stained with 0.1% Coomassie Brilliant Blue R-250 Dye (#20278 Thermo Fisher Scientific, USA) in 10% acetic acid, 50% methanol and 40% H_2_O for 16 h, and destained with destaining buffer (10% acetic acid, 50% methanol and 40% H_2_O) by soaking the gel for at least 2 h with at least two replacements of fresh solvent. Each lane was then individually removed from the gel and subjected to in-gel trypsin digestion after reduction with dithiothreitol and alkylation with iodoacetamide at Mass Spectrometry Facility, University of Massachusetts Medical School. Peptides eluted from the gel were lyophilized and re-suspended in 25µL of 5% acetonitrile (0.1% (v/v) trifluoroacetic acid). A 2-µL injection was loaded by a Waters NanoAcquity Ultra Performance Liquid Chromatography in 5% acetonitrile (0.1% formic acid) at 4.0 µL/min for 4 min onto a 100-µm I.D. fused-silica pre-column packed with 2 cm of 5 µm (200Å) Magic C18AQ (Bruker-Michrom Inc., MA, USA). Peptides were eluted at 300nL/min from a 75-µm I.D. gravity-pulled analytical column packed with 25 cm of 3 µm (100Å) Magic C18AQ particles using a linear gradient from 5-35% of mobile phase B (acetonitrile + 0.1% formic acid) in mobile phase A (water + 0.1% formic acid) over 60 min. Ions were introduced by positive electrospray ionization via liquid junction at 1.4kV into a Thermo Scientific Q Exactive hybrid mass spectrometer. Mass spectra were acquired over *m/z* 300-1750 at 70,000 resolution (*m/z* 200) with an AGC target of 1e6, and data-dependent acquisition selected the top 10 most abundant precursor ions for tandem mass spectrometry by higher energy collisional dissociation (HCD) fragmentation using an isolation width of 1.6 Da, max fill time of 110 ms, and AGC target of 1e^5^. Peptides were fragmented by a normalized collisional energy of 27, and fragment spectra acquired at a resolution of 17,500 (*m/z* 200).

### Mass-spectrometry data analysis

Raw data files were peak processed with Proteome Discoverer (version 1.4, Thermo Scientific, USA) followed by identification using Mascot Server (version 2.5, Matrix Science, UK) against the *Homo sapiens* (Swiss-Prot) FASTA file (downloaded 10/2018). Search parameters included Trypsin/P specificity, up to 2 missed cleavages, a fixed modification of carbamidomethyl cysteine, and variable modifications of oxidized methionine, pyroglutamic acid for Q, and N-terminal acetylation. Assignments were made using a 10ppm mass tolerance for the precursor and 0.05 Da mass tolerance for the fragments. All non-filtered search results were processed by Scaffold (version 4.4.4, Proteome Software, Inc., USA) utilizing the Trans-Proteomic Pipeline (Institute for Systems Biology, WA, USA) with threshold values set at 86% for peptides (1.0% false-discovery rate [FDR]) and 90% for proteins (2 peptide minimum, 0.2% FDR), and quantitative comparisons made using the iBAQ-quantitation method with all samples normalized by total ion current for the run [34].

#### Measurement of total tau (t-tau) and tau phosphorylated on threonine 181 (p-tau_181_)

The qEV fractions #7 and #8 were pooled, and 200μL of the pooled EVs was added to 5 volumes of ice cold 100% acetone to precipitate EV proteins. After a 16 h incubation at -20°C, the sample was spun at 20,000× *g* for 15 min at 4°C, and the pellet was dried up. The pellet was lysed with M-PER® Mammalian Protein Extraction Reagent (#78503 Thermo Fisher Scientific, USA) with Halt™ Protease and Phosphatase Inhibitor Cocktail (#78442 Thermo Fisher Scientific, USA) by vortexing for 15min. The EV t-tau and p-tau_181_ were measured using the modified Human Total Tau Simoa kit (Quanterix, Lexington, MA) on the Simoa HD-1 analyzer (Quanterix). Briefly, this kit is an updated version of the Simoa assay. It uses a monoclonal capture antibody that reacts with an epitope in the mid-region of all tau isoforms in combination with a detection antibody that reacts with an epitope at the N-terminus of t-tau for t-tau immunoassay or in paired helical filament (PHF)-tau (AT270, Thermo Fisher Scientific, USA) for p-tau_181_ immunoassay. The standards of t-tau or p-tau_181_ were adapted from Human Tau ELISA™ kit or Human Tau [pT181] phosphoELISA™ ELISA kit (Invitrogen, Thermo Fisher Scientific, USA) for each assay, respectively [35]. All plasma-derived EV samples were diluted 4x with the Tau 2.0 Sample Diluent (Invitrogen, Thermo Fisher Scientific, USA) prior to the assays, to minimize matrix effects, and were analyzed in duplicate on one occasion. The relative concentration estimates of t-tau and p-tau_181_ were calculated according to the standard curve.

**Table 1:** Patient information.

Proteomics	Control (n=12)	Former NFL player (n=14)	*t*-test^a^	*p*-value^b^
Age, mean	55.08 ± 6.42	56.71 ± 7.43	-0.594	0.558
Body Mass Index (BMI), mean	27.58 ± 3.31	33.44 ± 4.60	-3.669	0.001
Duration of football play, mean year	-	18.93 ± 4.10	-	-
Years in NFL, mean year	-	8.64 ± 3.48	-	-
Estimated cumulative head impacts in football^c^	-	20674.06 ± 6990.55	-	-
Mood / Behavior, mean	-0.821 ± 0.402	0.759 ± 1.048	-4.893	0.00006
Psychomotor speed / Executive Function, mean	0.430 ± 0.635	-0.189 ± 0.751	2.217	0.037
Verbal Memory, mean	0.506 ± 1.232	-0.248 ± 1.122	1.603	0.123
Visual Memory, mean	0.248 ± 0.752	-0.218 ± 0.784	1.515	0.144
**Ultrasensitive immunoassay for t-tau and p-tau**	**Control (n=25)**	**Former NFL player (n=27)**	***t*-test**	***p*-value**
Age, mean	57.04 ± 6.63	56.63 ± 7.60	0.207	0.837
Body Mass Index (BMI), mean	27.93 ± 3.77	33.50 ± 4.16	5.038	0.000007
Duration of football play, mean year	-	18.96 ± 3.65	-	-
Years in NFL, mean year	-	8.37 ± 3.00	-	-
Estimated cumulative head impacts in football^c^	-	19730.78 ± 5851.98	-	-
Mood / Behavior, mean	-0.920 ± 0.443	0.362 ± 0.771	-7.134	0.000000006
Psychomotor speed / Executive Function, mean	0.282 ± 0.650	-0.234 ± 0.728	2.588	0.013
Verbal Memory, mean	0.302 ± 1.193	-0.218 ± 0.788	1.794	0.084
Visual Memory, mean	0.329 ± 0.741	0.214 ± 0.761	0.525	0.602

a The group comparisons were performed using independent t-test.

b The statistical significance of the differences were calculated using a two-tailed test.

c Referred to as the Cumulative Head Impact Index (CHII) [11].

### Statistical Analysis

Statistical analysis was conducted using IBM SPSS software ver.25 and GraphPad Prism8 (GraphPad Software, CA, USA). Data were analyzed by t-test or, when appropriate, a nonparametric Mann-Whitney t-test. Group comparisons of age, body mass index (BMI), duration of football play, years in NFL, and the four clinical factor scores were performed using independent *t*-tests. The Gene Ontology of identified proteins were elucidated by the Database for Annotation, Visualization and Integrated Discovery (DAVID) Bioinformatics Resources 6.8 [36,37]. The Venn diagram and heatmap analysis were generated using Venny_2.1 (http://bioinfogp.cnb.csic.es/tools/venny/) and ClustVis (https://biit.cs.ut.ee/clustvis/).

### Machine Learning Analysis

An ensemble machine learning classifier to evaluate the performance of the selected proteins was developed [24,38]. The ensemble machine learning classifier consists of three individual machine learning algorithms to mitigate overfitting, including Linear Discriminant Analysis, Naïve Bayes and Support Vector Machine [38,39]. Model’s performance generated by the machine learning was evaluated on a separate, user-blinded test set (n = 26).

**Figure 1.**
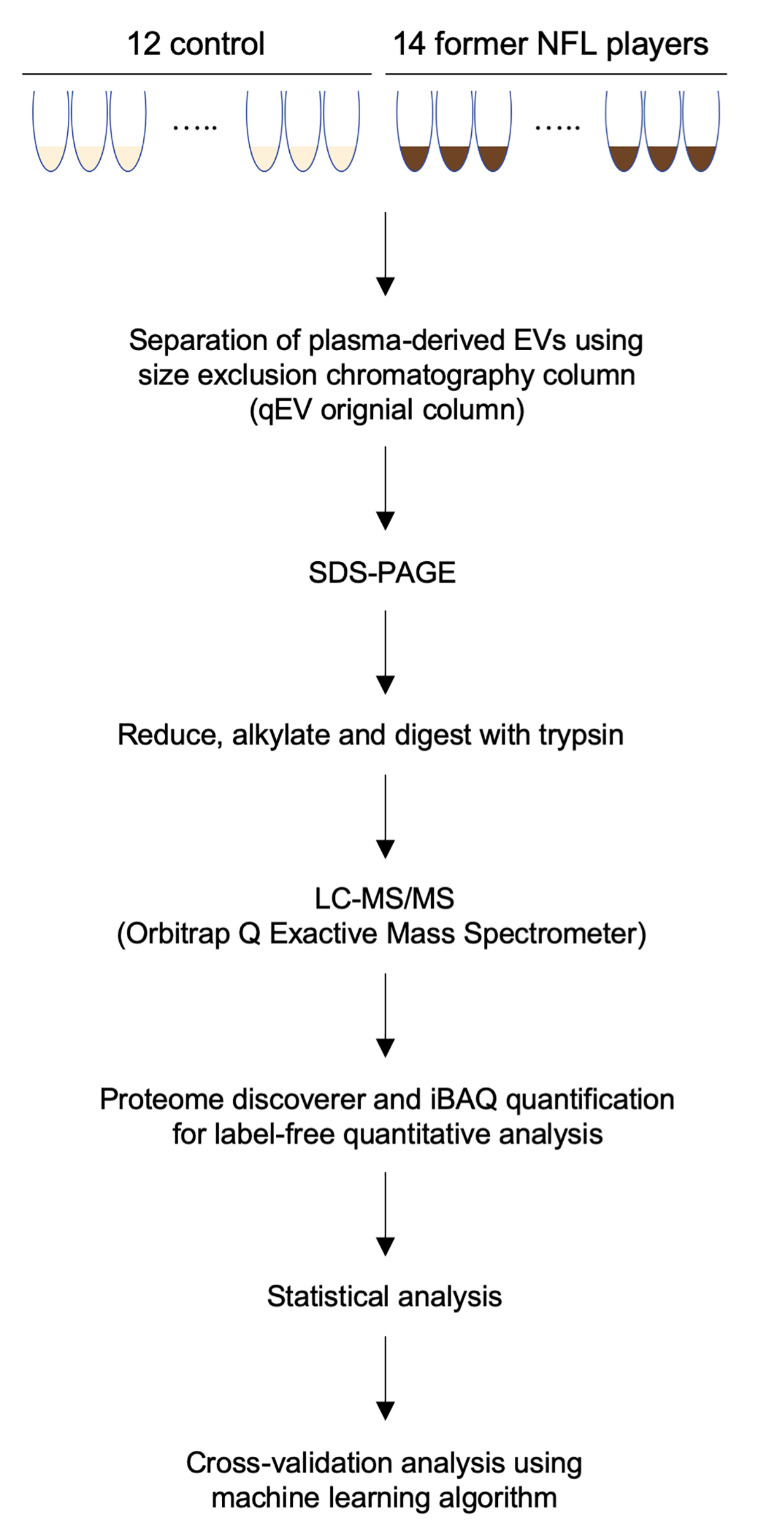
**Workflow used in proteomics analysis of former NFL player plasma-derived EVs**. EVs were separated from healthy control and former NFL players plasma using qEV original columns. The separated EVs were precipitated by acetone and run on 10% SDS-PAGE gel. The protein bands were cut out of the gel, followed by reduction, alkylation and trypsin digestion. The non-labeled peptides were analyzed by Nano LC-MS/MS on Orbitrap Q Exactive Mass Spectrometer. The raw data files were processed with Proteome Discoverer. Search results were loaded into the Scaffold Viewer to validate and quantify proteins. Biomarker candidate EV proteins for early diagnosis and monitoring of CTE were selected using bioinformatics analysis, and then confirmed the accuracy by Machine Learning algorithm.

## RESULTS

### Participants

Plasma samples were available for 26 participants, including 14 symptomatic former NFL players (mean age: 56.7 years, range 46-67) and 12 asymptomatic controls (mean age: 55.1, range 48-65) for proteomics, and 27 symptomatic former NFL players (mean age: 56.6, range 40-68) and 25 asymptomatic controls (mean age: 57.0, range 45-68) for ultrasensitive immunoassay for t-tau and p-tau (Table 1). There was statistical difference in body mass index (BMI) between former NFL players and control groups but no outlier was detected for BMI in the two groups as determined by Outlier Identifier with ROUT (Q = 1%) (Prism 8, GraphPad).

**Figure 2.**
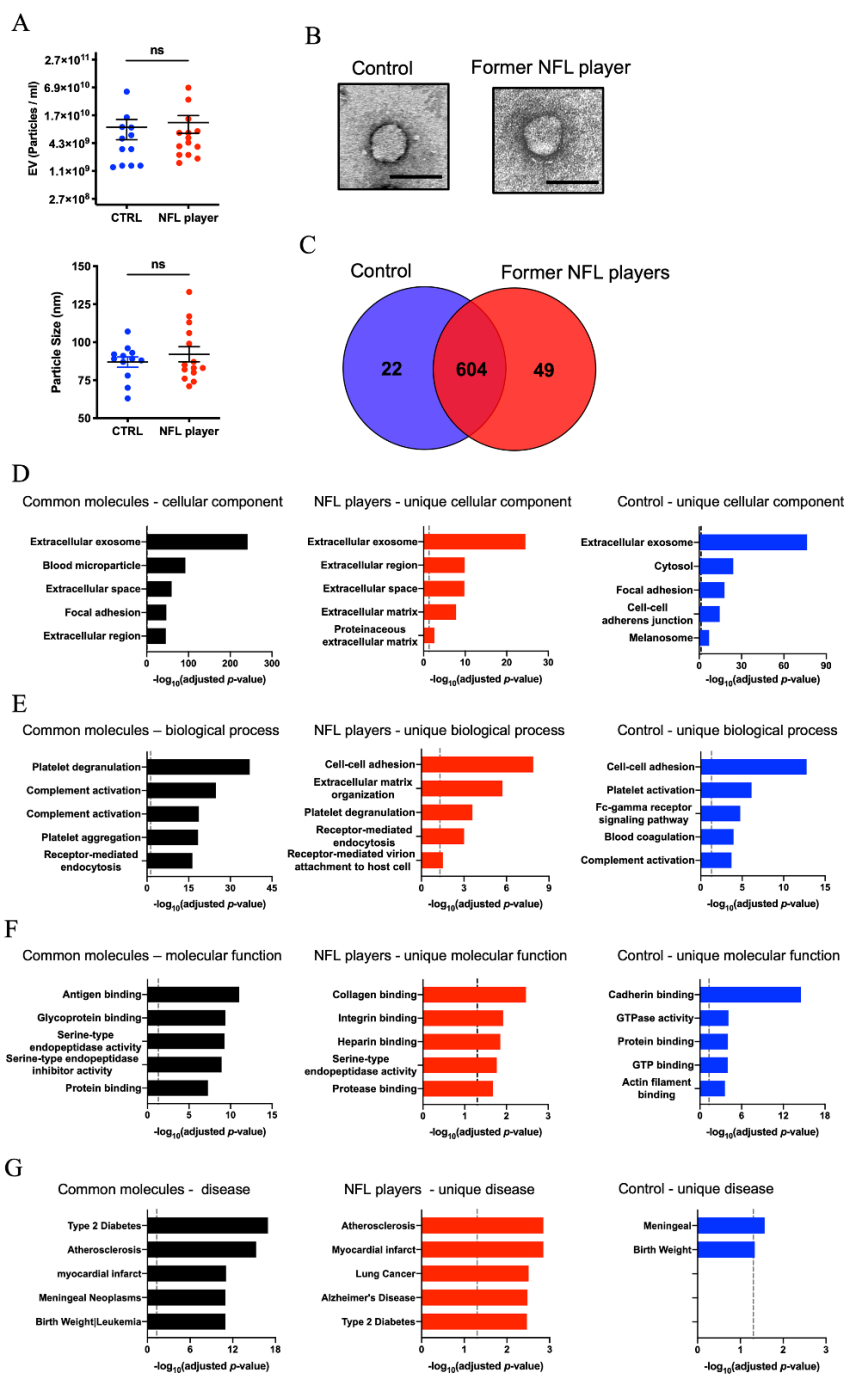
**Proteomic profiling of former NFL players and controls plasma-derived EVs**. (**A**) Upper panel: Particle numbers of Plasma-derived EV fraction from CTRLs and former NFL players by NTA (*p* = 0.4855 by Mann-Whitney test). Y-axis, log_2_ scale. Lower panel: Particle size of Plasma-derived EV fraction (*p* = 0.9497). (**B**) Transmission electron microscopy (TEM) image of former NFL player and control plasma-derived EV. Left: Control, Right: former NFL player. Scale bar; 100nm. (**C**) Venn diagram of the proteins identified in plasma-derived EVs from CTRLs (blue) and former NFL players (red). (**D-G**) Gene Ontology (GO) analysis using DAVID Bioinformatics Resources 6.8. (**D**) The GO term of Top 5 Cellular Component with -log_10_(FDR *p*-value). (**E**) The GO term of Top 5 Biological Process with -log_10_(FDR *p*-value). (**F**) The GO term of Top 5 Molecular Function with -log_10_(FDR *p*-value). (**G**) The GO term of Top 5 Disease Ontology with -log_10_(FDR *p*-value).

### Experimental workflow

The experimental workflow is summarized in Figure 1. The EVs were separated using qEV columns. For proteome profiling, the separated EVs were run in SDS-PAGE for in-gel digestion. The digested peptides were then analyzed by high sensitivity mass spectrometry (see Materials and Methods).

### Biochemical characteristic of plasma-derived EVs

NTA revealed that the concentrations of particles derived from former NFL players and controls were not significantly different (*p* = 0.4855). The mode size distribution peaked at 89 nm in former NFL players and at 84 nm in controls (*p* = 0.9497) (Fig. 2A). In addition, the separated EVs from former NFL player and control plasma were showed classical EV morphology by transmission electron microscopy (TEM, Fig. 2B).

**Figure 3.**
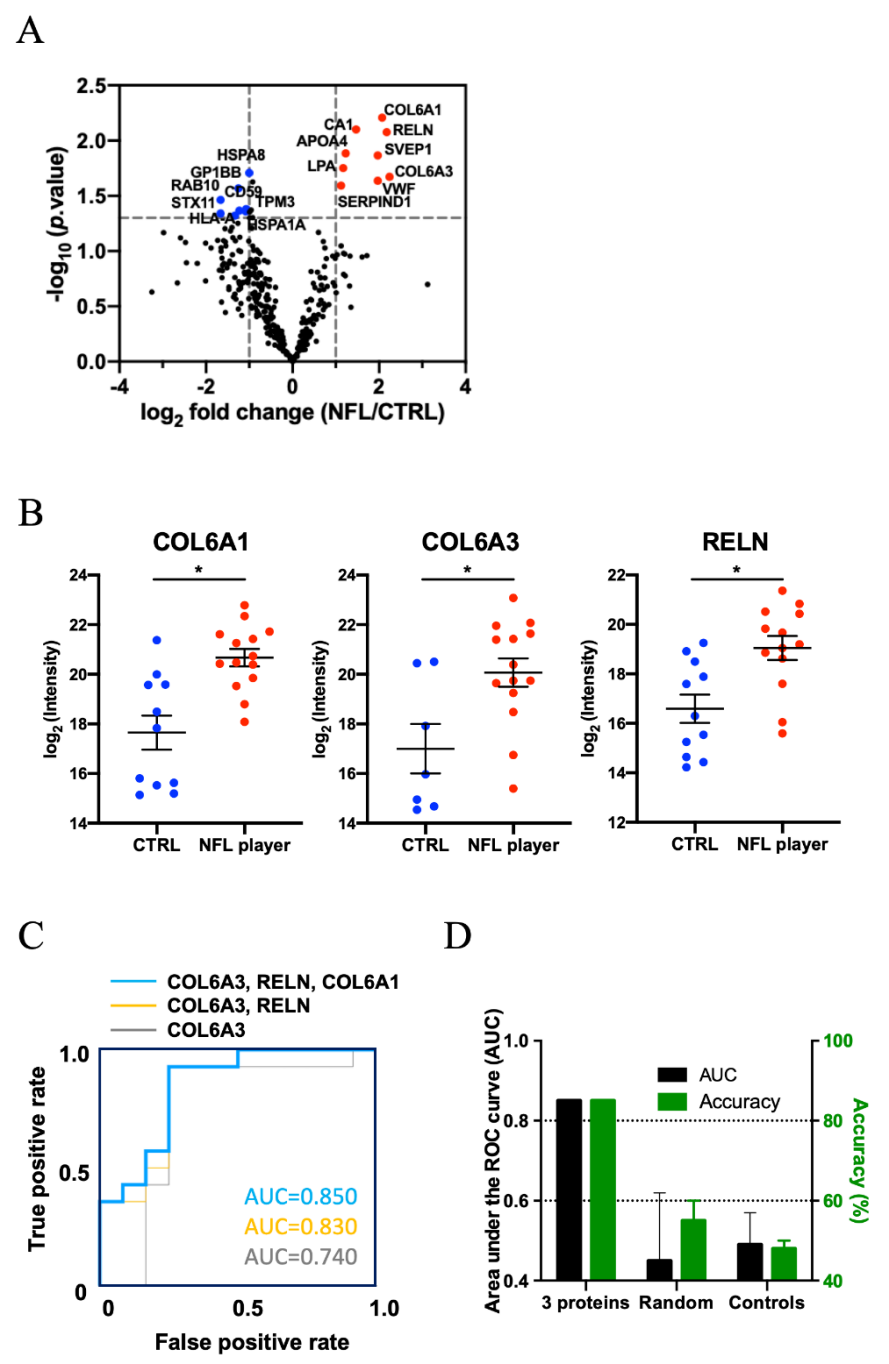
**Analysis of label-free quantitative proteomics comparison of former NFL players and control plasma-derived EV**. (**A**) Volcano plot showing degree of differential expression of EV proteins in former NFL players compared with CTRLs. The x-axis indicates log_2_ transformed fold change in expression. The y-axis shows -log_10_ transformed p-values. The grey dot line shows the 1.3010 -log_10_(*p*-value) and 1 or -1 log_2_(fold change) cutoff. (**B**) A scatter plot of log_2_ (intensity) as measured by proteomics per selected candidate protein. (COL6A1: -log_10_(*p*-value) = 2.2079, log_2_(intensity) = 2.07, COL6A3: -log_10_(*p*-value) = 1.6715, log_2_(intensity) = 2.24, RELN: -log_10_(*p*-value) = 2.0766, log_2_(intensity) = 2.18. The t test was calculated by Mann-Whitney test. (**C**) A ROC of possible pairs of three candidate proteins. Area under ROC for single-marker (COL6A3; Gray line) was 0.74, in multi-marker (COL6A3 and RELN; Orange line) were 0.83 and in multi-marker (COL6A3, RELN and COL6A1; Blue line) were 0.85. (**D**) The Accuracy for 3 selected using the test set: Accuracy = 85%, AUC = 0.85, Randomly selected control: Accuracy = 55%, AUC = 0.45, Shuffling control: Accuracy = 48%, AUC = 0.49.

### Proteomics profiling of former NFL player and control plasma-derived EVs

Across both groups, a total of 675 proteins were identified with at least two unique peptides (Fig. 2C and Supplementary Table 1 and 2). There were 626 proteins identified in the control group EVs and 653 proteins identified in former NFL player group EVs. Among them, 604 proteins were identified in both groups, with 22 proteins unique to the controls and 49 proteins unique to the former NFL players (Fig. 2C). The identified EV proteins were tested for gene ontology by the DAVID (Fig. 2D-G). Across all three groups (controls, former NFL players, and common proteins) the majority of expressed proteins were annotated as being components of the extracellular exosome category, a subset of the ‘cellular component’ ontology (Fig. 2D). Receptor-mediated endocytosis was among the 5 most enriched ‘biological process’ in EVs isolated from plasma of NFL players (Fig. 2E). Collagen and integrin binding activity was enriched in ‘molecular function’ GO terms in NFL players (Fig. 2F). In the ‘disease’ ontology, the molecules unique to former NFL player EVs were enriched in the AD category, but not in the control EVs (Fig. 2G). The EV samples were enriched in EV-specific molecules, such as tetraspanins (CD9 and CD81), annexins, Rab family, and also contain non-EV molecules such as APOA1, APOB and ALB, as listed in MISEV2018 guidelines [40] (Supplementary Table 3). Outlier identifier with “Robust nonlinear regression and Outlier removal method” detected no outlier among tested groups for APOA1 and ALB and one outlier for APOB, demonstrating that there was no obvious outlier in terms of contaminated proteins in these samples.[Table T2-ad-12-6-1363]

**Table 2 T2-ad-12-6-1363:** Up- and down-regulated plasma-derived EV proteins in former NFL players compared with controls.

Uniprot ID	Gene Name	Control Average Intensity^a^	Former NFL Player Average Intensity	log_2_ (NFL / Control)	*p*.value^b^	Count (control)^c^	Count (NFL)
P12111	COL6A3	4.73E+05	2.23E+06	2.24	0.0213	7	14
P78509-3	RELN	1.97E+05	8.90E+05	2.18	0.0084	11	13
P12109	COL6A1	5.61E+05	2.35E+06	2.07	0.0062	11	14
Q4LDE5	SVEP1	1.09E+05	4.30E+05	1.97	0.0136	7	12
P04275	VWF	1.19E+07	4.67E+07	1.97	0.0230	12	14
P00915	CA1	9.83E+05	2.72E+06	1.47	0.0079	9	14
P06727	APOA4	1.12E+07	2.64E+07	1.23	0.0131	12	14
P08519	LPA	4.52E+07	1.02E+08	1.17	0.0177	12	14
P05546	SERPIND1	7.41E+05	1.61E+06	1.12	0.0255	10	13
P11142	HSPA8	1.20E+07	6.01E+06	-1.00	0.0197	11	14
P13987	CD59	6.82E+06	3.26E+06	-1.07	0.0416	10	11
P0DMV8-2	HSPA1A	3.89E+06	1.84E+06	-1.08	0.0438	9	13
P06753-5	TPM3	1.78E+07	7.60E+06	-1.23	0.0430	11	14
P13224-2	GP1BB	4.44E+07	1.87E+07	-1.25	0.0271	12	14
P04439	HLA-A	3.06E+07	1.22E+07	-1.33	0.0478	12	14
P61026	RAB10	1.87E+07	5.93E+06	-1.66	0.0344	10	11
O75558	STX11	3.69E+06	1.17E+06	-1.66	0.0457	9	11

a The value shows iBAQ intensity by Scaffold software.

b The statistical significance of the differences were calculated using student’s t.test.

c The count shows identified patient numbers.

### Potential of plasma-derived EV proteins as a biomarker for CTE

**Figure 4.**
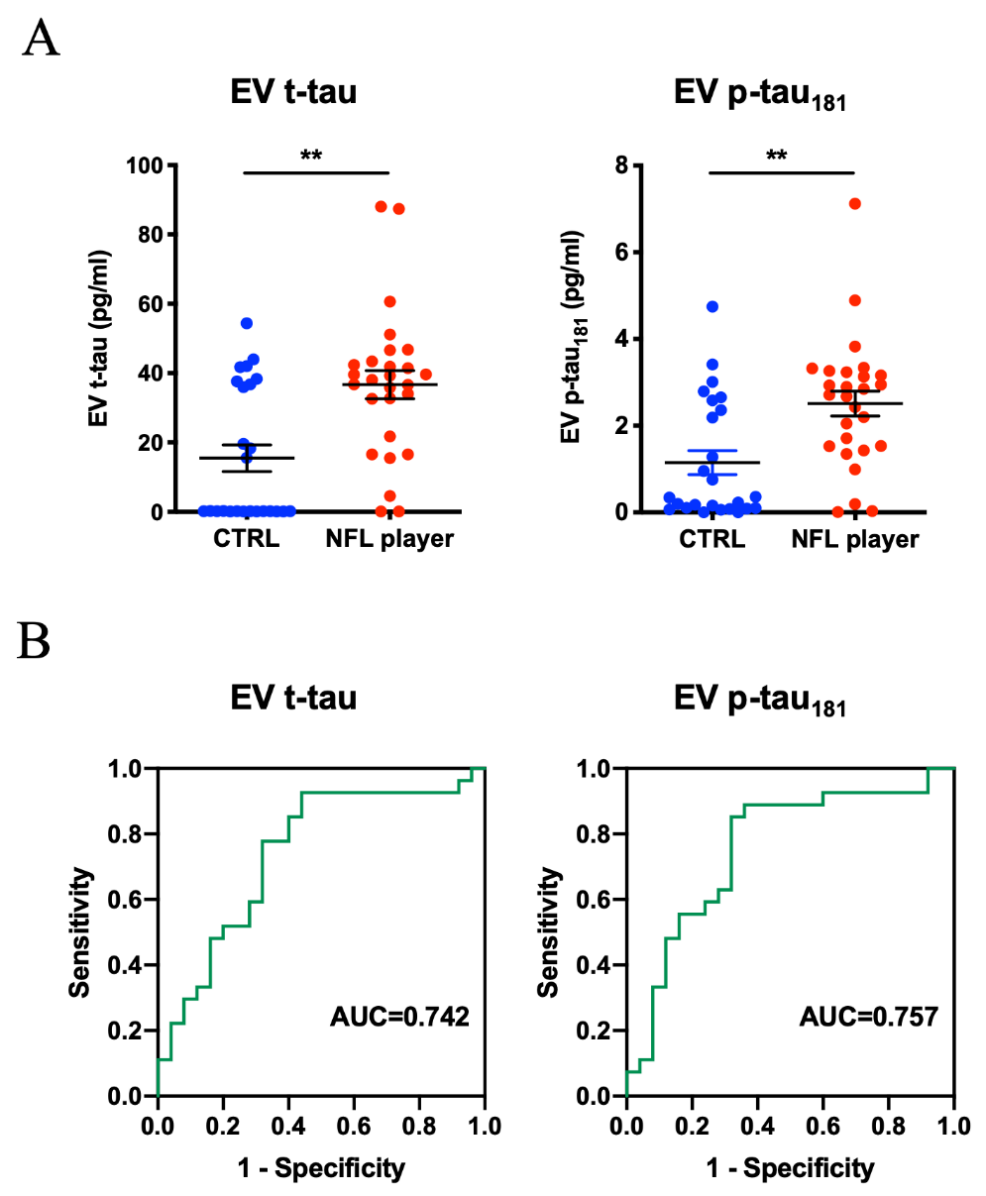
**Levels of Plasma-derived EV t-tau and p-tau from former NFL players and controls**. (**A**) Total-tau and tau phosphorylated at threonine 181 (p-tau_181_) levels in Plasma-derived EVs in the other cohort (former NFL players = 27, CTRL = 25) by ultrasensitive immunoassay. Left: EV t-tau levels (*p* = 0.0022). Right: EV p-tau_181_ levels (*p* = 0.0011). (**B**) The ROC curves of EV t-tau and p-tau_181._ Left: EV t-tau ROC curve (AUC = 0.742). Right: EV p-tau_181_ ROC (AUC = 0.757).

Label-free quantitative comparisons were performed using the iBAQ-quantification method within the Scaffold software. Figure 3A shows the volcano plot of the 325 common proteins which were detected in more than 50% of the group (NFL players: n > 6 and controls: n > 5). Among these proteins, 9 proteins were significantly up-regulated and 8 proteins were significantly down-regulated in former NFL players compared to the controls (as determined by -log_10_(*p*-value) <1.30 and log_2_ fold-change threshold of >1 or <-1) (Fig. 3A, Supplementary Fig. 1A and Table 2). Figure 3B shows the scatter plot of top 3 proteins (Collagen type VI alpha 3 and 1 chain (COL6A3 and COL6A1), reelin (RELN), which were significantly differentially expressed between NFL players and control groups. We assessed the correlation of these 17 proteins with the clinical factor score by Spearman’s correlation analysis (Supplementary Fig. 1A and B). To determine the combination of the proteins with the highest receiver operating characteristic (ROC), the significantly differentially expressed proteins were analyzed using an ensemble machine learning algorithm. The AUC value for the combination of the top 3 up-regulated molecules (COL6A3, RELN and COL6A1) was significantly higher than the combination of the top 2 up-regulated molecules or using only the single most up-regulated molecule (Fig. 3C and D). The combination of the top 3 up-regulated molecules exhibited 85% accuracy in identifying former NFL players (AUC = 0.85). In confirmation, we ran two control experiments that randomly selected 3 proteins to form the identification panel (repeated 20 times, AUC = 0.45, accuracy = 55%) and shuffling the true labels of the subjects within the training set (AUC = 0.49, accuracy = 48%). Neither approached the level of accuracy of the initial finding (Fig. 3C and D). To characterize the CTE-related proteins in plasma-derived EVs, we measured the concentration of t-tau and p-tau_181_ in the other cohort by ultrasensitive immunoassay. Both t-tau and p-tau_181_ were detectable in former NFL players’ plasma-derived EVs, and the levels of each were significantly different (t-tau: *p* = 0.0022, p-tau_181_: *p* = 0.0011, Fig. 4A). ROC curve was performed to assess the biomarker potential of t-tau and p-tau_181_ in plasma-derived EVs. The t-tau and p-tau_181_ in plasma-derived EVs showed an AUC of 0.742 and 0.757, respectively (Fig. 4B). We assessed the ROC and AUC value for combination of t-tau or p-tau_181_ and significantly differentially expressed proteins (Supplementary Table 4). The combinations of t-tau or p-tau_181_ and CA1 or STX11 were significantly higher than using only the single t-tau or p-tau_181_ molecule (Supplementary Fig. 2).

## DISCUSSION

EVs separated from plasma of former NFL players with symptoms consistent with CTE and same-age asymptomatic controls underwent label-free quantitative proteomic profiling by Nano LC-MS/MS. We identified 675 proteins across both groups, 17 of which show significant differences in expression between former NFL players and controls. Machine learning analysis revealed that the combination of COL6A3, RELN and COL6A1 proteins distinguished the former NFL players from controls with 85% accuracy. These molecules are associated with severity of neuropsychiatric features, but are not associated with the duration of football. We have also identified 49 unique molecules in the former NFL players, which show unique association with atherosclerosis, myocardial infarction, lung cancer, and AD as determined by DAVID GO.

Gene ontology analysis revealed that enrichment of extracellular exosomes between two groups, suggesting that this size-exclusion chromatography method can significantly enrich EVs from plasma sample. Interestingly, disease analysis of the EVs showed NFL players enriched proteins related to AD, which is another tauopathy. Moreover, NFL player plasma EVs were enriched in receptor-mediated endocytosis in biological process, which are integral part of microglial function for the clearance of degenerated neurons and protein accumulations [41].

Accumulating evidence suggests that plasma- or serum-derived EVs may harbor pathogenic proteins or molecular information reflective of a neurodegenerative disorder, and therefore may assess the severity of sports-related mild traumatic brain injury (mTBI) and predict progression of CTE. Winston *et al* has reported that the levels of postsynaptic protein neurogranin in plasma neuron-derived EVs (NDE) and astrocyte- derived EVs (ADE) were significantly lower in mTBI patients and their plasma NDEs cargo protein are toxic to recipient cells [42]. Goetzl *et al* has described that the level of neurofunctional protein, including annexin VII, UCH-L1, occludin, aquaporin 4 and synaptogyrin-3, in plasma NDE were elevated in the acute mTBI phase but mostly normalized in chronic phase while they observed sustained abnormal increase in pathogenic proteins, such as Aβ42 and p-tau_181_, in chronic mTBI patients [43]. Our proteomics data was identified only annexin Vll (fold change = 0.71, *p*-value = 0.5241), but it tends toward decrease in NFL players.

The protein levels of COL6A3, RELN and COL6A1 were significantly increased in the EVs in the plasma of former NFL players. Previous research found that sciatic crush injury increases the expression level of *Col6a1*, and *Col6a1*^-/-^ mice show delayed regeneration of sciatic nerve with reduced macrophage recruitment and their polarization towards M2 neurodegenerative phenotype [44], suggesting that M2 macrophage-derived EVs may contribute to NFL players’ plasma EV to some degree. Moreover, our recent study shows white matter rarefaction as a result of repeated head impacts in former NFL players, which may be more relatable to the expression of COL6A3 and COL6A1 rather than the pTau pathology of CTE. Reelin protein has been reported to be up-regulated in the brains of AD patients with advanced Braak stage [45]. In the healthy brain, reelin binds to apolipoprotein E receptor 2 (ApoER2) as an active homodimer and induces phosphorylation of Dab1 [45,46]. The phosphorylated Dab1 prevents tau phosphorylation. In advanced AD, Aβ increases reelin levels and further disrupts active homodimers [45,47-49]. Thus, increased reelin observed in NFL players at risk of CTE may suggest increase in tau phosphorylation due to altered reelin signaling. COL6A1, COL6A3 and reelin are all extracellular matrix proteins and may interact with integrins enriched in EVs. The EV contents of these molecules may be increased in advanced CTE brain in due neurodegeneration, which would release these molecules in part of the cell death. The brain-derived EVs loaded with these molecule may be exported to plasma and detected in our proteomic profiling.

In our previous report that exosomal tau is elevated in former NFL players with risk of CTE, we purified plasma EV by size-exclusion chromatography and detected EV-associated tau using anti-human tau monoclonal antibody and Qdot-655-conjugated secondary antibody [30], which might not detect tau within EVs and was less quantitative. There was also a general concern about the reproducibility of NTA-based detection of florescence-conjugated EVs, which is less sensitive due to the limitation in the laser power for the wide-field fluorescence microscopic imaging. In this study, we isolated plasma EVs using qEV and quantified t-tau and p-tau_181_ after solubilization of EVs by highly sensitive and quantitative Simoa technology, and confirmed that the level of both t-tau and p-tau_181_ were significantly increased in former NFL players compared to controls. We have previously found no differences in plasma t-tau levels between the former NFL players and controls [11]. One explanation for this finding is that we used different method for the quantification of the total tau. In the paper of Alosco *et al.* [11], t-tau was measured with the Simoa HD-1 analyzer (Quanterix) using human Tau kits (101444). In this study, we used modified kit using uses a monoclonal capture antibody that reacts with an epitope in the mid-region of all tau isoforms in combination with a detection antibody that reacts with an epitope at the N-terminus of t-tau for t-tau immunoassay or in paired helical filament-tau (AT270) for p-tau_181_ immunoassay. Our data indicate that plasma contains N-terminal truncated of tau, which was undetectable by the standard SIMOA Tau kits. Indeed, tau can be cleaved by various proteases, including calpain-1 and 2 at R230, thrombin, cathepsins, asparagine endopeptidase at D255 and N368 [50,51]. This may have resulted in detection of a higher level of t-tau by modified kit over standard kit. The EV tau in plasma may therefore represent a non-invasive biomarker for monitoring the progression of CTE. Importantly, the ROC and AUC value revealed that the AUC of the combinations of COL6A3, RELN, and COL6A1 were significantly higher than AUC of the t-tau or p-tau_181_ alone.

Across both former NFL players and controls, a total of 675 proteins were identified, with 22 proteins unique to the controls and 49 proteins unique to the former NFL players. Although the proteins that were unique to each group are expected as biomarker candidate molecules, there are several limitations in this study. First, they have only been identified in a small number of former NFL players or control groups. Second, not all former NFL players would be expected to have CTE and those with CTE likely are at different stages of p-tau pathology and neurodegeneration. Third, there is a possibility of other neurodegenerative diseases in both groups, and within the former NFL group, there is a possibility of concurrent proteinopathies with CTE. Fourth, recent studies suggest that outcome of cognitive function with former NFL players may be affected by existing comorbidities such as atherosclerosis, higher BMI, white matter rarefaction or other proteinopathies including Lewy body disease [10,52,53]. Indeed, we observed significant negative correlation between BMI and Psychomotor speed/ Executive Function values when both CTRL and NFL players are combined, although there was no correlation when two groups are separately analyzed, which is a potential confounding factor in this study. Aging is also a confounding factor of psychomotor speed / executive Function [54]. However, there was no correlation between age and Psychomotor speed / Executive Function values in this study. Finally, the candidate proteins may only distinguish former NFL players from controls regardless of the cognitive status. Thus, it is plausible for us to replicate this study with larger samples, controlling for these variables and, ultimately, perform post-mortem validation to determine if these proteins serve as potential biomarkers in former NFL players at risk for CTE.

In summary, this study presents the first proteomic profiles of plasma-derived EVs from symptomatic former NFL players at risk for the neurodegenerative tauopathy, CTE. At this time, CTE is diagnosed through postmortem neuropathological examination. There is a need for relatively non-invasive in vivo biomarkers for the diagnosis and early detection of CTE. Our results suggest that a combination of COL6A1, COL6A3 and RELN molecules in plasma-derived EVs may meet this need. It’s still unclear whether these proteins can be used as brain-derived EVs in plasma as COL6A1 and COL6A3 are commonly expressed in multiple tissues. The combination with cell type-specific molecules from brain cells may serve as more suitable brain-specific biomarkers for CTE. Further investigations are necessary to confirm the utility of this approach, including additional validation sets for monitoring of the disease progression, and to clarify the implications of the correlation of these markers with the disease, as well as to elucidate the mechanisms of EV transport from the brain to plasma.

## Supplementary Materials

The Supplemenantry data can be found online at: www.aginganddisease.org/EN/10.14336/AD.2020.0908.


