# Supplementary material for: Proteomic Profiling of Extracellular Vesicles Separated from Plasma of Former National Football League Players at Risk for Chronic Traumatic Encephalopathy
Source: Aging Dis. 2021 Sep 1;12(6):1363–75. doi: 10.14336/AD.2020.0908 (PMC8407879; doi:10.14336/AD.2020.0908)
Supplement: Supplementary file 1 [file AD-12-6-1363-s.pdf]

SUPPLEMENTARY DATA

Proteomic Profiling of Extracellular Vesicles Separated from Plasma of Former National Football League Players at Risk for Chronic Traumatic Encephalopathy

Satoshi Muraoka[1], Annina M. DeLeo[1], Zijian Yang[2], Harutsugu Tatebe[3], Kayo Yukawa-Takamatsu[1], Seiko Ikezu[1], Takahiko Tokuda[3], David Issadore[2], Robert A. Stern[4,5], Tsuneya Ikezu[1,4,6*]

© 2020. Muraoka S. et al. Published online at http://www.aginganddisease.org/EN/10.14336/AD.2020.0908

SUPPLEMENTARY DATA

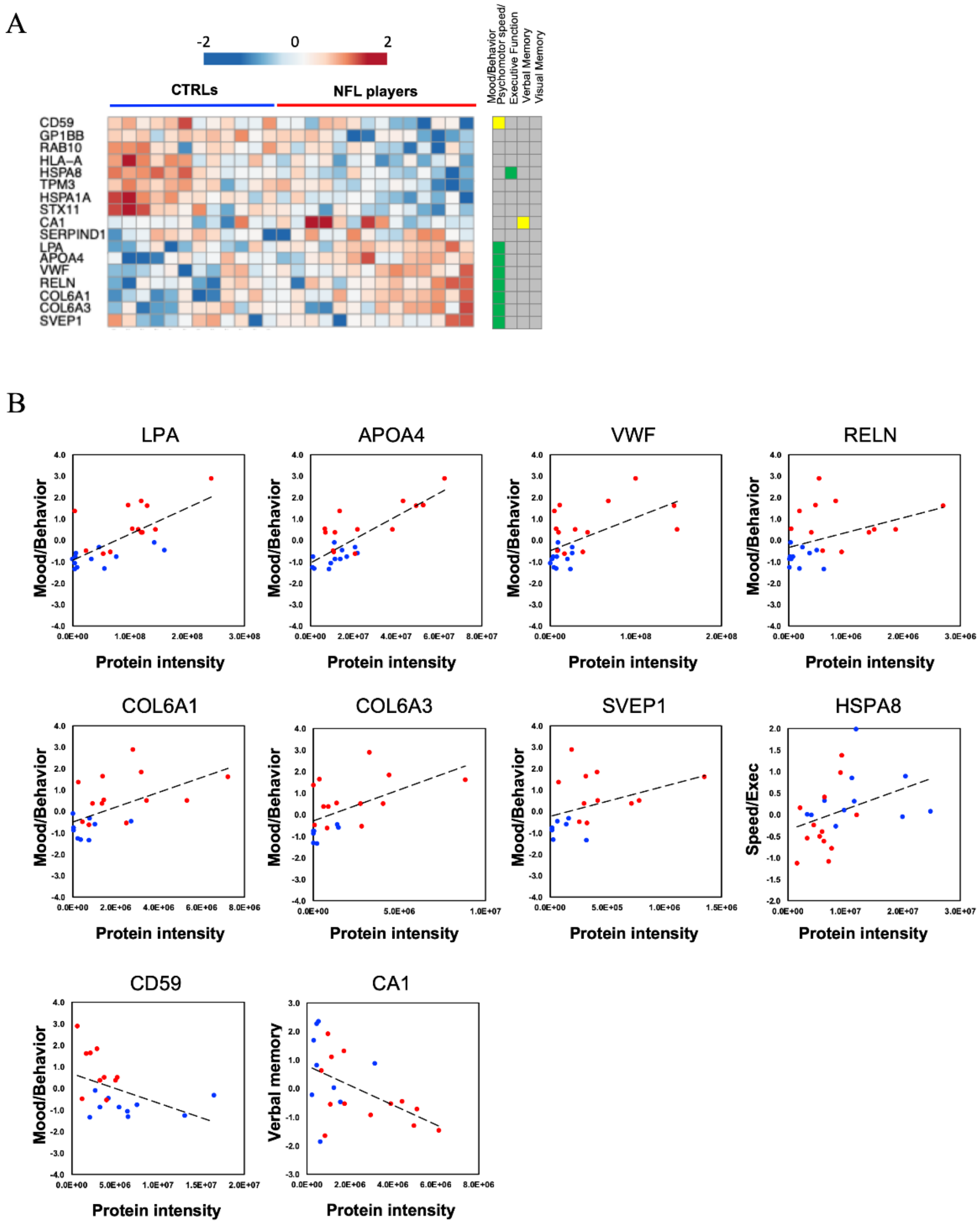

Supplementary Figure 1. Association of these 17 proteins with the clinical factor scores: **A)** Heatmap of 17 proteins with the 1.3010 -log10(p-value) and 1 or -1 log2 transformed fold change cutoff. The value shows zscore. The heatmap on the far right indicates correlation of 17 proteins with the clinical factor scores. Green color shows positive correlation. Yellow color shows negative correlation. Gray color shows no significant correlation. **B)** Scatter plot of CD59, LPA, APOA4, VWF, RELN, COL6A1, COL6A3 or SVEP1 and Mood/Behavior, CA1 and Verbal Memory, and HSPA8 and Speed Exec. Positive correlation of LPA with Mood/Behavior (rho = 0.663, $p < 0.001$), APOA4 with Mood/Behavior (rho = 0.630, p = 0.001), VWF with Mood/Behavior (rho = 0.523, p = 0.007), RELN with Mood/Behavior (rho = 0.489, p = 0.018), COL6A1 with Mood/Behavior (rho = 0.657, $p < 0.001$), COL6A3 with Mood/Behavior (rho = 0.630, p = 0.003), SVEP1 with Mood/Behavior (rho = 0.505, p = 0.033), HSPA8 with Speed/Exec (rho = 0.477, p = 0.019). Negative correlation of CD59 with Mood/Behavior (rho = -0.471, p = 0.036), CA1 with Verbal Memory (rho = -0.464, p = 0.030). Red dot indicates former NFL players. Blue dot indicates CTRL groups.

© 2020. Muraoka S. et al. Published online at http://www.aginganddisease.org/EN/10.14336/AD.2020.0908

SUPPLEMENTARY DATA

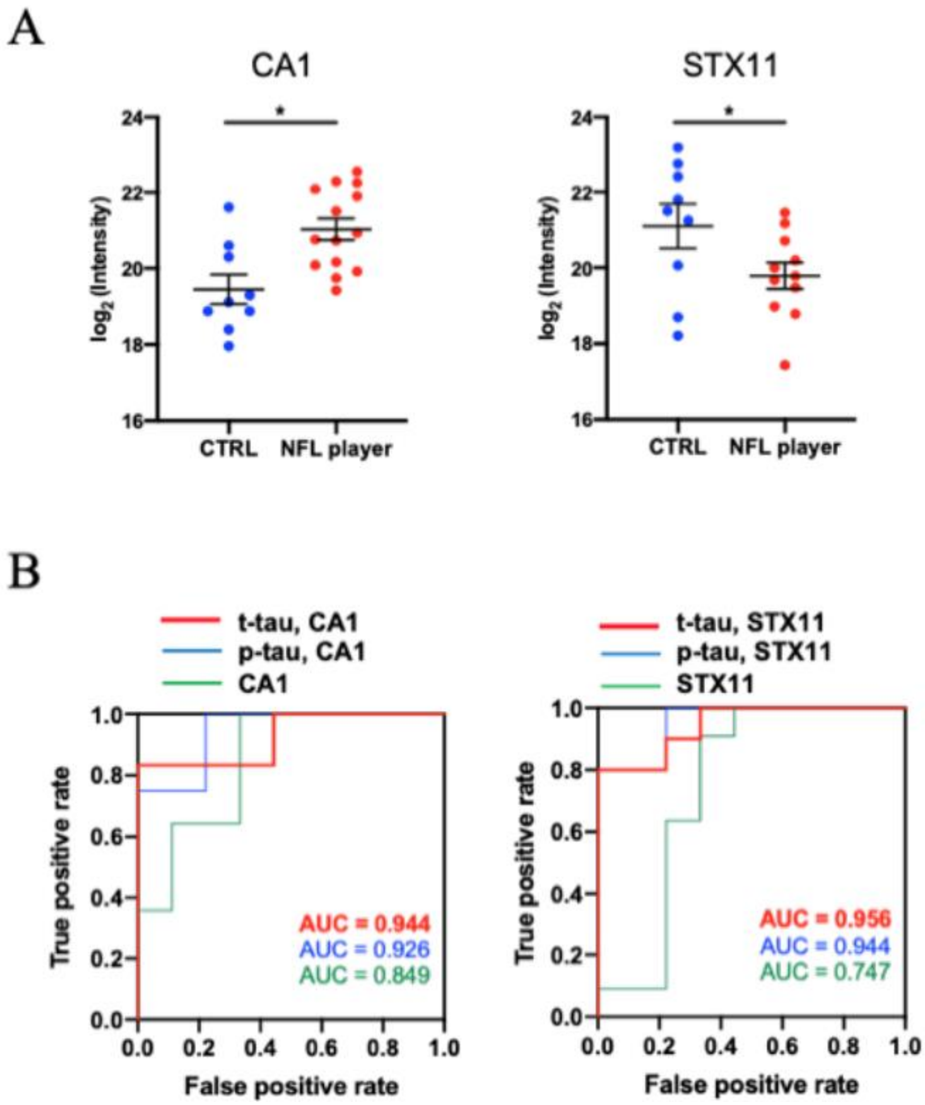

Supplementary Figure 2. Potential biomarkers for former NFL players at risk for CTE: **A)** A scatter plot of log2 (intensity) as measured by proteomics per selected candidate protein. (CA1: -log10(p-value) = 2.1018, log2(fold change) = 1.47, STX11: -log10(p-value) = 1.3405, log2(fold change) = -1.66). The t test was calculated by Mann-Whitney test. **B)** The ROC curves of possible pairs of EV t-tau or p-tau181 and CA1 or STX11 candidate proteins. Left: CA1: AUC for CA1 single-marker (CA1; Green line) was 0.849, in multi-marker (t-tau and CA1; Red line) was 0.944 and in multi-marker (p-tau181 and CA1; Blue line) was 0.926. Right: STX11: AUC for STX11 single-marker (STX11; Green line) was 0.747, in multi-marker (t-tau and STX11; Red line) was 0.956 and in multi-marker (p-tau181 and STX11; Blue line) was 0.944.

© 2020. Muraoka S. et al. Published online at http://www.aginganddisease.org/EN/10.14336/AD.2020.0908

SUPPLEMENTARY DATA

Supplementary Table 1. Number of Protein Identification in Plasma-derived EV proteomics dataset.

Sample ID	Protein identification number[a]	Sample ID	Protein identification number
Control_1	445	Former NFL player_1	354
Control_2	448	Former NFL player_2	318
Control_3	465	Former NFL player_3	307
Control_4	208	Former NFL player_4	499
Control_5	377	Former NFL player_5	303
Control_6	419	Former NFL player_6	519
Control_7	390	Former NFL player_7	481
Control_8	310	Former NFL player_8	378
Control_9	410	Former NFL player_9	375
Control_10	315	Former NFL player_10	340
Control_11	411	Former NFL player_11	502
Control_12	343	Former NFL player_12	451
		Former NFL player_13	415
		Former NFL player_14	359

[a]The number of proteins were identified by LC-MS/MS.

Supplementary Table 2. Identification and quantification of EVs isolated from former NFL players and controls plasma.

Number	Uniprot ID	Gene name	Description	Molecular Weight	Intensity average (control groups)[a]	Intensity average (former NFL players)	Fold change (NFL/control)	*p*-value[b]
1	P04114	APOB	APOB_HUMAN Apolipoprotein B-100	516 kDa	5.27E+08	9.21E+08	1.75	0.2085
2	P02768	ALB	ALBU_HUMAN Serum albumin	69 kDa	2.27E+09	3.41E+09	1.50	0.1552
3	P13645	KRT10	K1C10_HUMAN Keratin, type I cytoskeletal 10	59 kDa	5.13E+08	4.88E+08	0.95	0.8986
4	Q9Y490	TLN1	TLN1_HUMAN Talin-1	270 kDa	5.35E+07	2.79E+07	0.52	0.0621
5	P21333-2	FLNA	FLNA_HUMAN Isoform 2 of Filamin-A	280 kDa	4.43E+07	2.34E+07	0.53	0.0903
6	P35908	KRT2	K22E_HUMAN Keratin, type II cytoskeletal 2 epidermal	65 kDa	3.45E+08	2.94E+08	0.85	0.7073
7	P01023	A2M	A2MG_HUMAN Alpha-2-macroglobulin	163 kDa	1.35E+08	1.60E+08	1.18	0.7823
8	P11277-2	SPTB	SPTB1_HUMAN Isoform 2 of Spectrin beta chain, erythrocytic	268 kDa	1.32E+07	1.51E+07	1.14	0.7446
9	P02549	SPTA1	SPTA1_HUMAN Spectrin alpha chain, erythrocytic 1	280 kDa	1.16E+07	1.41E+07	1.21	0.6233
10	P04275	VWF	VWF_HUMAN von Willebrand factor	309 kDa	1.19E+07	4.67E+07	3.92	0.0230

© 2020. Muraoka S. et al. Published online at http://www.aginganddisease.org/EN/10.14336/AD.2020.0908

SUPPLEMENTARY DATA

11	P68032	ACTC1	ACTC_HUMAN Actin, alpha cardiac muscle 1	42 kDa	4.58E+08	2.91E+08	0.64	0.1216
12	P01024	C3	CO3_HUMAN Complement C3	187 kDa	1.13E+07	1.47E+07	1.30	0.3396
13	P35527	KRT9	K1C9_HUMAN Keratin, type I cytoskeletal 9	62 kDa	5.63E+08	3.06E+08	0.54	0.2622
14	P04264	KRT1	K2C1_HUMAN Keratin, type II cytoskeletal 1	66 kDa	1.01E+09	5.80E+08	0.57	0.2623
15	P02649	APOE	APOE_HUMAN Apolipoprotein E	36 kDa	6.88E+08	1.12E+09	1.63	0.0820
16	P02787	TF	TRFE_HUMAN Serotransferrin	77 kDa	7.14E+07	8.36E+07	1.17	0.5350
17	P16157-16	ANK1	ANK1_HUMAN Isoform Er15 of Ankyrin-1	204 kDa	1.15E+07	1.61E+07	1.40	0.4593
18	P08519	LPA	APOA_HUMAN Apolipoprotein(a)	501 kDa	4.52E+07	1.02E+08	2.26	0.0177
19	P08514	ITGA2B	ITA2B_HUMAN Integrin alpha-IIb	113 kDa	1.37E+08	9.21E+07	0.67	0.1254
20	P18206-2	VCL	VINC_HUMAN Isoform 1 of Vinculin	117 kDa	2.58E+07	1.38E+07	0.54	0.0984
21	P02647	APOA1	APOA1_HUMAN Apolipoprotein A-I	31 kDa	3.75E+08	4.22E+08	1.12	0.6681
22	P02730	SLC4A1	B3AT_HUMAN Band 3 anion transport protein	102 kDa	1.11E+08	1.37E+08	1.23	0.5594
23	P02671	FGA	FIBA_HUMAN Fibrinogen alpha chain	95 kDa	4.16E+07	7.46E+07	1.79	0.3047
24	P02675	FGB	FIBB_HUMAN Fibrinogen beta chain	56 kDa	5.83E+07	1.15E+08	1.98	0.2065
25	Q86UX7-2	FERMT3	URP2_HUMAN Isoform 2 of Fermitin family homolog 3	75 kDa	5.78E+07	3.27E+07	0.57	0.0979
26	P69905	HBA1	HBA_HUMAN Hemoglobin subunit alpha	15 kDa	1.62E+09	1.40E+09	0.86	0.6889
27	P02679-2	FGG	FIBG_HUMAN Isoform Gamma-A of Fibrinogen gamma chain	49 kDa	1.04E+08	1.71E+08	1.64	0.2991
28	P00739-2	HPR	HPTR_HUMAN Isoform 2 of Haptoglobin-related protein	43 kDa	7.06E+07	1.65E+08	2.33	0.1669
29	Q9H4B7	TUBB1	TBB1_HUMAN Tubulin beta-1 chain	50 kDa	3.24E+07	1.95E+07	0.60	0.1643
30	P63104	YWHAZ	1433Z_HUMAN 14-3-3 protein zeta/delta	28 kDa	1.21E+08	4.61E+07	0.38	0.1080
31	P35579	MYH9	MYH9_HUMAN Myosin-9	227 kDa	2.82E+06	3.00E+06	1.06	0.8897
32	P01871	IGHM	IGHM_HUMAN Immunoglobulin heavy constant mu	49 kDa	1.66E+09	1.34E+09	0.81	0.6415
33	P12259	F5	FA5_HUMAN Coagulation factor V	252 kDa	4.34E+06	4.35E+06	1.00	0.9893
34	P05106	ITGB3	ITB3_HUMAN Integrin beta-3	87 kDa	5.02E+07	2.98E+07	0.59	0.1051
35	P26038	MSN	MOES_HUMAN Moesin	68 kDa	1.48E+07	1.73E+07	1.17	0.4438
36	Q9Y6R7	FCGBP	FCGBP_HUMAN IgGFc-binding protein	572 kDa	3.39E+06	7.82E+06	2.31	0.1065
37	P01009	SERPINA1	A1AT_HUMAN Alpha-1-antitrypsin	47 kDa	4.20E+07	6.37E+07	1.51	0.0677
38	Q71U36-2	TUBA1A	TBA1A_HUMAN Isoform 2 of Tubulin alpha-1A chain	46 kDa	3.42E+07	2.09E+07	0.61	0.1964
39	P23229-9	ITGA6	ITA6_HUMAN Isoform 9 of Integrin alpha-6	122 kDa	8.27E+06	5.51E+06	0.67	0.2486
40	P04003	C4BPA	C4BPA_HUMAN C4b-binding protein alpha chain	67 kDa	3.11E+07	4.72E+07	1.52	0.3742
41	P12111	COL6A3	CO6A3_HUMAN Collagen alpha-3(VI) chain	344 kDa	4.73E+05	2.23E+06	4.72	0.0213
42	P06727	APOA4	APOA4_HUMAN Apolipoprotein A-IV	45 kDa	1.12E+07	2.64E+07	2.35	0.0131
43	O75636	FCN3	FCN3_HUMAN Ficolin-3	33 kDa	4.13E+08	1.61E+08	0.39	0.2604
44	P06396-2	GSN	GELS_HUMAN Isoform 2 of Gelsolin	81 kDa	1.38E+07	1.03E+07	0.74	0.3220

© 2020. Muraoka S. et al. Published online at http://www.aginganddisease.org/EN/10.14336/AD.2020.0908

SUPPLEMENTARY DATA

45	P01834	IGKC	IGKC_HUMAN Immunoglobulin kappa constant	12 kDa	1.54E+09	1.27E+09	0.82	0.6958
46	P15924	DSP	DESP_HUMAN Desmoplakin	332 kDa	2.34E+06	4.23E+05	0.18	0.0835
47	P06753-5	TPM3	TPM3_HUMAN Isoform 5 of Tropomyosin alpha-3 chain	29 kDa	1.78E+07	7.60E+06	0.43	0.0430
48	P14618	PKM	KPYM_HUMAN Pyruvate kinase PKM	58 kDa	1.53E+07	8.08E+06	0.53	0.0236
49	P02786	TFRC	TFR1_HUMAN Transferrin receptor protein 1	85 kDa	1.01E+07	9.97E+06	0.99	0.9803
50	P68871	HBB	HBB_HUMAN Hemoglobin subunit beta	16 kDa	1.67E+09	2.09E+09	1.25	0.5052
51	P04040	CAT	CATA_HUMAN Catalase	60 kDa	1.55E+07	1.74E+07	1.12	0.7024
52	P0C0L5	C4B	CO4B_HUMAN Complement C4-B	193 kDa	1.73E+06	2.41E+06	1.40	0.1965
53	P02751-14	FN1	FINC_HUMAN Isoform 14 of Fibronectin	249 kDa	1.22E+06	4.00E+06	3.29	0.1100
54	P0DOY2	IGLC2	IGLC2_HUMAN Immunoglobulin lambda constant 2	11 kDa	2.55E+08	2.27E+08	0.89	0.8044
55	P61224	RAP1B	RAP1B_HUMAN Ras-related protein Rap-1b	21 kDa	2.01E+08	1.01E+08	0.50	0.0499
56	P11171-2	EPB41	41_HUMAN Isoform 2 of Protein 4.1	93 kDa	5.37E+06	6.67E+06	1.24	0.6030
57	Q08380	LGALS3BP	LG3BP_HUMAN Galectin-3-binding protein	65 kDa	2.47E+07	6.14E+07	2.49	0.2066
58	P00488	F13A1	F13A_HUMAN Coagulation factor XIII A chain	83 kDa	1.01E+07	1.18E+07	1.17	0.6974
59	Q9HBI1-3	PARVB	PARVB_HUMAN Isoform 3 of Beta-parvin	38 kDa	4.90E+07	2.49E+07	0.51	0.1210
60	P27105	STOM	STOM_HUMAN Erythrocyte band 7 integral membrane protein	32 kDa	5.59E+07	3.87E+07	0.69	0.1811
61	P24821-4	TNC	TENA_HUMAN Isoform 4 of Tenascin	231 kDa	1.20E+06	2.73E+06	2.27	0.1621
62	P55056	APOC4	APOC4_HUMAN Apolipoprotein C-IV	15 kDa	7.31E+08	1.02E+09	1.40	0.4059
63	P06733	ENO1	ENOA_HUMAN Alpha-enolase	47 kDa	1.29E+07	7.59E+06	0.59	0.1578
64	A1L4H1	SSC5D	SRCRL_HUMAN Soluble scavenger receptor cysteine-rich domain-containing protein SSC5D	166 kDa	3.87E+06	6.65E+06	1.72	0.1169
65	P12814-3	ACTN1	ACTN1_HUMAN Isoform 3 of Alpha-actinin-1	106 kDa	2.96E+06	3.73E+06	1.26	0.6958
66	P04406-2	GAPDH	G3P_HUMAN Isoform 2 of Glyceraldehyde-3-phosphate dehydrogenase	32 kDa	4.06E+07	3.10E+07	0.76	0.2132
67	P02790	HPX	HEMO_HUMAN Hemopexin	52 kDa	1.49E+07	2.07E+07	1.39	0.3962
68	P04899	GNAI2	GNAI2_HUMAN Guanine nucleotide-binding protein G(i) subunit alpha-2	40 kDa	1.96E+07	1.50E+07	0.76	0.2847
69	P07195	LDHB	LDHB_HUMAN L-lactate dehydrogenase B chain	37 kDa	1.76E+07	9.55E+06	0.54	0.1279
70	P01876	IGHA1	IGHA1_HUMAN Immunoglobulin heavy constant alpha 1	38 kDa	1.41E+08	2.17E+08	1.53	0.2265
71	Q9UHG3	PCYOX1	PCYOX_HUMAN Prenylcysteine oxidase 1	57 kDa	9.94E+06	2.50E+07	2.52	0.1107
72	P07996	THBS1	TSP1_HUMAN Thrombospondin-1	129 kDa	4.40E+06	4.47E+06	1.02	0.9756
73	P10909-5	CLU	CLUS_HUMAN Isoform 5 of Clusterin	54 kDa	2.65E+07	2.89E+07	1.09	0.7586
74	P55072	VCP	TERA_HUMAN Transitional endoplasmic reticulum ATPase	89 kDa	6.01E+06	7.32E+06	1.22	0.7783
75	Q13418	ILK	ILK_HUMAN Integrin-linked protein kinase	51 kDa	1.14E+07	7.73E+06	0.68	0.3996
76	P16671	CD36	CD36_HUMAN Platelet glycoprotein 4	53 kDa	4.30E+07	2.93E+07	0.68	0.2721

© 2020. Muraoka S. et al. Published online at http://www.aginganddisease.org/EN/10.14336/AD.2020.0908

77	P01042-2	KNG1	KNG1_HUMAN Isoform LMW of Kininogen-1	48 kDa	4.04E+07	2.75E+07	0.68	0.6836
78	P12931-2	SRC	SRC_HUMAN Isoform 2 of Proto-oncogene tyrosine-protein kinase Src	61 kDa	7.70E+06	3.84E+06	0.50	0.1669
79	P11142	HSPA8	HSP7C_HUMAN Heat shock cognate 71 kDa protein	71 kDa	1.20E+07	6.01E+06	0.50	0.0197
80	P00450	CP	CERU_HUMAN Ceruloplasmin	122 kDa	2.23E+06	3.51E+06	1.57	0.1751
81	P07359	GP1BA	GP1BA_HUMAN Platelet glycoprotein Ib alpha chain	72 kDa	4.30E+07	2.17E+07	0.50	0.0442
82	O43866	CD5L	CD5L_HUMAN CD5 antigen-like	38 kDa	5.08E+07	3.99E+07	0.78	0.5990
83	P05556	ITGB1	ITB1_HUMAN Integrin beta-1	88 kDa	1.35E+07	8.07E+06	0.60	0.1584
84	P27169	PON1	PON1_HUMAN Serum paraoxonase/arylesterase 1	40 kDa	2.28E+07	3.79E+07	1.66	0.1376
85	Q7L576	CYFIP1	CYFP1_HUMAN Cytoplasmic FMR1-interacting protein 1	145 kDa	2.26E+06	1.12E+06	0.49	0.1801
86	P07225	PROS1	PROS_HUMAN Vitamin K-dependent protein S	75 kDa	3.98E+06	6.47E+06	1.63	0.3914
87	P40197	GP5	GPV_HUMAN Platelet glycoprotein V	61 kDa	1.46E+07	6.07E+06	0.42	0.0560
88	P16284-3	PECAM1	PECA1_HUMAN Isoform Delta13 of Platelet endothelial cell adhesion molecule	80 kDa	5.26E+06	2.70E+06	0.51	0.1365
89	P16452-2	EPB42	EPB42_HUMAN Isoform Long of Erythrocyte membrane protein band 4.2	80 kDa	4.42E+06	4.51E+06	1.02	0.9602
90	P00736	C1R	C1R_HUMAN Complement C1r subcomponent	80 kDa	4.61E+06	3.95E+06	0.86	0.7598
91	P03951	F11	FA11_HUMAN Coagulation factor XI	70 kDa	1.18E+07	8.85E+06	0.75	0.7084
92	P05090	APOD	APOD_HUMAN Apolipoprotein D	21 kDa	4.59E+07	7.70E+07	1.68	0.1747
93	P32119	PRDX2	PRDX2_HUMAN Peroxiredoxin-2	22 kDa	9.08E+07	6.66E+07	0.73	0.4641
94	P02749	APOH	APOH_HUMAN Beta-2-glycoprotein 1	38 kDa	1.31E+07	1.49E+07	1.14	0.5856
95	P02656	APOC3	APOC3_HUMAN Apolipoprotein C-III	11 kDa	1.40E+09	1.70E+09	1.22	0.7052
96	O75083	WDR1	WDR1_HUMAN WD repeat-containing protein 1	66 kDa	1.15E+07	4.42E+06	0.38	0.0755
97	P62937	PPIA	PPIA_HUMAN Peptidyl-prolyl cis-trans isomerase A	18 kDa	7.94E+07	2.15E+07	0.27	0.0937
98	P78509-3	RELN	RELN_HUMAN Isoform 3 of Reelin	384 kDa	1.97E+05	8.90E+05	4.53	0.0084
99	P07737	PFN1	PROF1_HUMAN Profilin-1	15 kDa	6.91E+07	4.58E+07	0.66	0.3166
100	Q00610-2	CLTC	CLH1_HUMAN Isoform 2 of Clathrin heavy chain 1	188 kDa	3.42E+05	7.77E+05	2.27	0.1048
101	P01008	SERPINC1	ANT3_HUMAN Antithrombin-III	53 kDa	3.40E+06	4.89E+06	1.44	0.2138
102	P08567	PLEK	PLEK_HUMAN Pleckstrin	40 kDa	2.03E+07	1.75E+07	0.86	0.6433
103	Q01518-2	CAP1	CAP1_HUMAN Isoform 2 of Adenylyl cyclase-associated protein 1	52 kDa	7.06E+06	3.50E+06	0.50	0.1557
104	Q15833-2	STXBP2	STXB2_HUMAN Isoform 2 of Syntaxin-binding protein 2	66 kDa	3.19E+06	1.80E+06	0.56	0.2700
105	P07900-2	HSP90AA1	HS90A_HUMAN Isoform 2 of Heat shock protein HSP 90-alpha	98 kDa	2.72E+06	1.55E+06	0.57	0.1195
106	P23528	CFL1	COF1_HUMAN Cofilin-1	19 kDa	6.37E+07	2.51E+07	0.39	0.1307
107	P02654	APOC1	APOC1_HUMAN Apolipoprotein C-I	9 kDa	5.23E+08	6.18E+08	1.18	0.6781

108	O14791-2	APOL1	APOL1_HUMAN Isoform 2 of Apolipoprotein L1	46 kDa	1.35E+07	1.15E+07	0.85	0.7363
109	P02774-3	GC	VTDB_HUMAN Isoform 3 of Vitamin D-binding protein	55 kDa	2.93E+06	3.89E+06	1.33	0.4240
110	P63000-2	RAC1	RAC1_HUMAN Isoform B of Ras-related C3 botulinum toxin substrate 1	23 kDa	2.29E+07	9.84E+06	0.43	0.0748
111	O95445	APOM	APOM_HUMAN Apolipoprotein M	21 kDa	5.30E+07	5.04E+07	0.95	0.8791
112	P04075	ALDOA	ALDOA_HUMAN Fructose-bisphosphate aldolase A	39 kDa	1.45E+07	5.79E+06	0.40	0.0807
113	P11166	SLC2A1	GTR1_HUMAN Solute carrier family 2, facilitated glucose transporter member 1	54 kDa	2.12E+07	2.48E+07	1.17	0.6478
114	P61026	RAB10	RAB10_HUMAN Ras-related protein Rab-10	23 kDa	1.87E+07	5.93E+06	0.32	0.0344
115	P04004	VTN	VTNC_HUMAN Vitronectin	54 kDa	3.99E+06	3.32E+06	0.83	0.6460
116	Q13201	MMRN1	MMRN1_HUMAN Multimerin-1	138 kDa	1.12E+06	7.47E+05	0.67	0.5538
117	P00491	PNP	PNPH_HUMAN Purine nucleoside phosphorylase	32 kDa	4.28E+06	2.42E+06	0.57	0.1754
118	P35542	SAA4	SAA4_HUMAN Serum amyloid A-4 protein	15 kDa	6.26E+07	7.07E+07	1.13	0.7949
119	P02655	APOC2	APOC2_HUMAN Apolipoprotein C-II	11 kDa	6.95E+08	8.62E+08	1.24	0.6161
120	P37802-2	TAGLN2	TAGL2_HUMAN Isoform 2 of Transgelin-2	24 kDa	1.88E+07	1.30E+07	0.69	0.5464
121	P01011	SERPINA 3	AACT_HUMAN Alpha-1-antichymotrypsin	48 kDa	6.08E+06	7.89E+06	1.30	0.4343
122	P30041	PRDX6	PRDX6_HUMAN Peroxiredoxin-6	25 kDa	1.73E+07	5.50E+06	0.32	0.1003
123	O00299	CLIC1	CLIC1_HUMAN Chloride intracellular channel protein 1	27 kDa	7.02E+06	5.30E+06	0.76	0.5423
124	P14923	JUP	PLAK_HUMAN Junction plakoglobin	82 kDa	4.75E+06	1.18E+06	0.25	0.0846
125	P04439	HLA-A	1A03_HUMAN HLA class I histocompatibility antigen, A-3 alpha chain	41 kDa	3.06E+07	1.22E+07	0.40	0.0478
126	P17301	ITGA2	ITA2_HUMAN Integrin alpha-2	129 kDa	1.39E+06	9.02E+05	0.65	0.1781
127	Q14644	RASA3	RASA3_HUMAN Ras GTPase-activating protein 3	96 kDa	2.25E+06	9.61E+05	0.43	0.1970
128	P00558	PGK1	PGK1_HUMAN Phosphoglycerate kinase 1	45 kDa	5.24E+06	2.62E+06	0.50	0.1274
129	P07355-2	ANXA2	ANXA2_HUMAN Isoform 2 of Annexin A2	40 kDa	1.89E+06	1.72E+06	0.91	0.8177
130	P04217	A1BG	A1BG_HUMAN Alpha-1B-glycoprotein	54 kDa	4.45E+06	5.84E+06	1.31	0.4211
131	P07384	CAPN1	CAN1_HUMAN Calpain-1 catalytic subunit	82 kDa	2.43E+06	1.31E+06	0.54	0.1389
132	Q9ULI3	HEG1	HEG1_HUMAN Protein HEG homolog 1	147 kDa	1.12E+06	2.23E+06	1.99	0.1161
133	P48059	LIMS1	LIMS1_HUMAN LIM and senescent cell antigen-like-containing domain protein 1	37 kDa	1.11E+07	4.06E+06	0.37	0.1230
134	Q4LDE5	SVEP1	SVEP1_HUMAN Sushi, von Willebrand factor type A, EGF and pentraxin domain-containing protein 1	390 kDa	1.09E+05	4.30E+05	3.93	0.0136
135	P30481	HLA-B	1B44_HUMAN HLA class I histocompatibility antigen, B-44 alpha chain	40 kDa	1.56E+07	1.02E+07	0.65	0.2622
136	P0DOX5		IGG1_HUMAN Immunoglobulin gamma-1 heavy chain	49 kDa	1.70E+08	2.86E+08	1.68	0.0405
137	P01859	IGHG2	IGHG2_HUMAN Immunoglobulin heavy constant gamma 2	36 kDa	1.15E+08	1.37E+08	1.19	0.4037

© 2020. Muraoka S. et al. Published online at http://www.aginganddisease.org/EN/10.14336/AD.2020.0908

138	P02042	HBD	HBD_HUMAN Hemoglobin subunit delta	16 kDa	6.47E+08	1.03E+09	1.59	0.2027
139	P02746	C1QB	C1QB_HUMAN Complement C1q subcomponent subunit B	27 kDa	1.43E+07	1.81E+07	1.26	0.5681
140	P08758	ANXA5	ANXA5_HUMAN Annexin A5	36 kDa	4.64E+06	1.49E+06	0.32	0.1155
141	P14770	GP9	GPIX_HUMAN Platelet glycoprotein IX	19 kDa	3.44E+07	1.95E+07	0.57	0.1985
142	P50148	GNAQ	GNAQ_HUMAN Guanine nucleotide-binding protein G(q) subunit alpha	42 kDa	6.83E+06	2.98E+06	0.44	0.1006
143	P61204	ARF3	ARF3_HUMAN ADP-ribosylation factor 3	21 kDa	1.34E+07	8.15E+06	0.61	0.2252
144	P00747	PLG	PLMN_HUMAN Plasminogen	91 kDa	1.03E+06	2.24E+06	2.17	0.0898
145	P50395	GDI2	GDIB_HUMAN Rab GDP dissociation inhibitor beta	51 kDa	1.77E+06	9.04E+05	0.51	0.1194
146	O14672	ADAM10	ADA10_HUMAN Disintegrin and metalloproteinase domain-containing protein 10	84 kDa	2.70E+06	1.25E+06	0.46	0.1556
147	P19827	ITIH1	ITIH1_HUMAN Inter-alpha-trypsin inhibitor heavy chain H1	101 kDa	1.05E+06	1.99E+06	1.91	0.1108
148	P08575-2	PTPRC	PTPRC_HUMAN Isoform 2 of Receptor-type tyrosine-protein phosphatase C	131 kDa	7.18E+05	9.41E+05	1.31	0.5957
149	Q15404	RSU1	RSU1_HUMAN Ras suppressor protein 1	32 kDa	1.74E+07	8.87E+06	0.51	0.1333
150	P02766	TTR	TTHY_HUMAN Transthyretin	16 kDa	2.58E+07	1.82E+07	0.71	0.3681
151	P12109	COL6A1	CO6A1_HUMAN Collagen alpha-1(VI) chain	109 kDa	5.61E+05	2.35E+06	4.20	0.0062
152	P61158	ACTR3	ARP3_HUMAN Actin-related protein 3	47 kDa	4.73E+06	2.27E+06	0.48	0.1564
153	P30740	SERPINB1	ILEU_HUMAN Leukocyte elastase inhibitor	43 kDa	5.53E+06	1.78E+06	0.32	0.1743
154	P21926	CD9	CD9_HUMAN CD9 antigen	25 kDa	1.11E+08	4.20E+07	0.38	0.0612
155	A0A0B4J1X5	IGHV3-74	HV374_HUMAN Immunoglobulin heavy variable 3-74	13 kDa	8.93E+07	6.16E+07	0.69	0.4810
156	P62873-2	GNB1	GBB1_HUMAN Isoform 2 of Guanine nucleotide-binding protein G(I)/G(S)/G(T) subunit beta-1	36 kDa	1.62E+07	8.71E+06	0.54	0.1975
157	P00734	F2	THRB_HUMAN Prothrombin	70 kDa	1.28E+06	2.09E+06	1.64	0.3394
158	P05546	SERPIND1	HEP2_HUMAN Heparin cofactor 2	57 kDa	7.41E+05	1.61E+06	2.17	0.0255
159	P04196	HRG	HRG_HUMAN Histidine-rich glycoprotein	60 kDa	4.49E+06	3.59E+06	0.80	0.7343
160	Q9ULV4-2	CORO1C	COR1C_HUMAN Isoform 2 of Coronin-1C	54 kDa	3.27E+06	1.89E+06	0.58	0.1011
161	P13798	APEH	ACPH_HUMAN Acylamino-acid-releasing enzyme	81 kDa	6.55E+05	7.18E+05	1.10	0.7428
162	P18428	LBP	LBP_HUMAN Lipopolysaccharide-binding protein	53 kDa	4.42E+06	4.76E+06	1.08	0.8998
163	Q8WUM4	PDCD6IP	PDC6I_HUMAN Programmed cell death 6-interacting protein	96 kDa	4.67E+05	6.16E+05	1.32	0.5355
164	P02776	PF4	PLF4_HUMAN Platelet factor 4	11 kDa	1.03E+08	9.34E+07	0.91	0.8612
165	P01591	JCHAIN	IGJ_HUMAN Immunoglobulin J chain	18 kDa	3.96E+07	5.33E+07	1.35	0.5648
166	P10644	PRKAR1A	KAP0_HUMAN cAMP-dependent protein kinase type I-alpha regulatory subunit	43 kDa	2.81E+06	1.27E+06	0.45	0.1382
167	P13224-2	GP1BB	GP1BB_HUMAN Isoform 2 of Platelet glycoprotein Ib beta chain	43 kDa	4.44E+07	1.87E+07	0.42	0.0271

© 2020. Muraoka S. et al. Published online at http://www.aginganddisease.org/EN/10.14336/AD.2020.0908

SUPPLEMENTARY DATA

168	Q14624	ITIH4	ITIH4_HUMAN Inter-alpha-trypsin inhibitor heavy chain H4	103 kDa	7.22E+05	1.02E+06	1.41	0.3803
169	Q9HC84	MUC5B	MUC5B_HUMAN Mucin-5B	596 kDa	9.66E+05	1.70E+05	0.18	0.1331
170	O75955	FLOT1	FLOT1_HUMAN Flotillin-1	47 kDa	9.73E+05	1.05E+06	1.08	0.8501
171	P02775	PPBP	CXCL7_HUMAN Platelet basic protein	14 kDa	1.27E+08	6.58E+07	0.52	0.1625
172	P15144	ANPEP	AMPN_HUMAN Aminopeptidase N	110 kDa	6.78E+05	5.88E+06	8.67	0.2006
173	P00751	CFB	CFAB_HUMAN Complement factor B	86 kDa	1.57E+06	1.22E+06	0.77	0.6107
174	P02763	ORM1	A1AG1_HUMAN Alpha-1-acid glycoprotein 1	24 kDa	1.59E+07	1.93E+07	1.21	0.5639
175	P20073-2	ANXA7	ANXA7_HUMAN Isoform 2 of Annexin A7	50 kDa	1.74E+06	1.23E+06	0.71	0.5241
176	Q12913	PTPRJ	PTPRJ_HUMAN Receptor-type tyrosine-protein phosphatase eta	146 kDa	1.23E+06	4.44E+05	0.36	0.1299
177	Q02413	DSG1	DSG1_HUMAN Desmoglein-1	114 kDa	6.21E+06	8.53E+05	0.14	0.0712
178	P61586	RHOA	RHOA_HUMAN Transforming protein RhoA	22 kDa	7.86E+06	2.43E+06	0.31	0.1483
179	P01019	AGT	ANGT_HUMAN Angiotensinogen	53 kDa	1.90E+06	2.38E+06	1.26	0.4310
180	P0DMV8-2	HSPA1A	HS71A_HUMAN Isoform 2 of Heat shock 70 kDa protein 1A	64 kDa	3.89E+06	1.84E+06	0.47	0.0438
181	O00194	RAB27B	RB27B_HUMAN Ras-related protein Rab-27B	25 kDa	1.07E+07	3.49E+06	0.33	0.1345
182	P50995-2	ANXA11	ANX11_HUMAN Isoform 2 of Annexin A11	51 kDa	1.49E+06	1.26E+06	0.85	0.7290
183	Q8N1N4	KRT78	K2C78_HUMAN Keratin, type II cytoskeletal 78	57 kDa	3.17E+06	1.41E+06	0.45	0.3828
184	P01619	IGKV3-20	KV320_HUMAN Immunoglobulin kappa variable 3-20	13 kDa	4.52E+07	1.15E+08	2.55	0.3222
185	O15144	ARPC2	ARPC2_HUMAN Actin-related protein 2/3 complex subunit 2	34 kDa	2.82E+06	1.54E+06	0.55	0.2439
186	P02743	APCS	SAMP_HUMAN Serum amyloid P-component	25 kDa	3.10E+06	1.59E+06	0.51	0.0425
187	Q92954-2	PRG4	PRG4_HUMAN Isoform B of Proteoglycan 4	147 kDa	1.83E+05	3.72E+05	2.04	0.1078
188	P13716	ALAD	HEM2_HUMAN Delta-aminolevulinic acid dehydratase	36 kDa	4.24E+06	3.89E+06	0.92	0.8370
189	O15143	ARPC1B	ARC1B_HUMAN Actin-related protein 2/3 complex subunit 1B	41 kDa	6.58E+06	1.99E+06	0.30	0.0799
190	P05155-2	SERPING1	IC1_HUMAN Isoform 2 of Plasma protease C1 inhibitor	50 kDa	3.19E+06	3.94E+06	1.24	0.4423
191	P62979	RPS27A	RS27A_HUMAN Ubiquitin-40S ribosomal protein S27a	18 kDa	1.26E+07	1.06E+07	0.84	0.5301
192	Q00013	MPP1	EM55_HUMAN 55 kDa erythrocyte membrane protein	52 kDa	1.23E+06	1.66E+06	1.35	0.2784
193	P60953	CDC42	CDC42_HUMAN Cell division control protein 42 homolog	21 kDa	7.33E+06	5.43E+06	0.74	0.5242
194	P78417	GSTO1	GSTO1_HUMAN Glutathione S-transferase omega-1	28 kDa	5.26E+06	1.95E+06	0.37	0.0657
195	P02747	C1QC	C1QC_HUMAN Complement C1q subcomponent subunit C	26 kDa	1.69E+07	2.06E+07	1.21	0.6755
196	P19652	ORM2	A1AG2_HUMAN Alpha-1-acid glycoprotein 2	24 kDa	7.61E+06	1.20E+07	1.58	0.2786

© 2020. Muraoka S. et al. Published online at http://www.aginganddisease.org/EN/10.14336/AD.2020.0908

SUPPLEMENTARY DATA

197	P19823	ITIH2	ITIH2_HUMAN Inter-alpha-trypsin inhibitor heavy chain H2	106 kDa	6.96E+05	1.33E+06	1.92	0.1935
198	P51149	RAB7A	RAB7A_HUMAN Ras-related protein Rab-7a	23 kDa	4.20E+06	1.96E+06	0.47	0.1610
199	O00560	SDCBP	SDCB1_HUMAN Syntenin-1	32 kDa	1.43E+06	2.36E+06	1.65	0.2217
200	A0A0C4DH38	IGHV5-51	HV551_HUMAN Immunoglobulin heavy variable 5-51	13 kDa	2.55E+07	2.47E+07	0.97	0.9557
201	P19086	GNAZ	GNAZ_HUMAN Guanine nucleotide-binding protein G(z) subunit alpha	41 kDa	2.60E+06	1.26E+06	0.49	0.1938
202	P02765	AHSG	FETUA_HUMAN Alpha-2-HS-glycoprotein	39 kDa	4.89E+06	6.02E+06	1.23	0.5221
203	P25311	AZGP1	ZA2G_HUMAN Zinc-alpha-2-glycoprotein	34 kDa	2.07E+06	1.68E+06	0.81	0.7323
204	Q9NZN3	EHD3	EHD3_HUMAN EH domain-containing protein 3	61 kDa	4.03E+06	2.08E+06	0.52	0.3136
205	P02652-2	APOA2	APOA2_HUMAN Apolipoprotein A-II mutant extension	12 kDa	1.02E+08	7.25E+07	0.71	0.3429
206	P50552	VASP	VASP_HUMAN Vasodilator-stimulated phosphoprotein	40 kDa	6.20E+06	2.07E+06	0.33	0.0806
207	P04632	CAPNS1	CPNS1_HUMAN Calpain small subunit 1	28 kDa	3.88E+06	1.93E+06	0.50	0.0813
208	P06744-2	GPI	G6PI_HUMAN Isoform 2 of Glucose-6-phosphate isomerase	64 kDa	1.05E+06	5.19E+05	0.50	0.2688
209	P52209	PGD	6PGD_HUMAN 6-phosphogluconate dehydrogenase, decarboxylating	53 kDa	1.41E+06	5.98E+05	0.42	0.1892
210	P60174	TPI1	TPIS_HUMAN Triosephosphate isomerase	31 kDa	4.45E+06	2.72E+06	0.61	0.3109
211	Q06830	PRDX1	PRDX1_HUMAN Peroxiredoxin-1	22 kDa	3.85E+07	1.89E+07	0.49	0.1612
212	P47756-2	CAPZB	CAPZB_HUMAN Isoform 2 of F-actin-capping protein subunit beta	31 kDa	5.46E+06	1.83E+06	0.34	0.1291
213	Q9Y2A7-2	NCKAP1	NCKP1_HUMAN Isoform 2 of Nck-associated protein 1	130 kDa	7.12E+05	3.22E+05	0.45	0.1936
214	P23467-3	PTPRB	PTPRB_HUMAN Isoform 3 of Receptor-type tyrosine-protein phosphatase beta	249 kDa	7.14E+04	1.66E+05	2.32	0.0922
215	P01860	IGHG3	IGHG3_HUMAN Immunoglobulin heavy constant gamma 3	41 kDa	9.72E+07	1.31E+08	1.34	0.1530
216	P43652	AFM	AFAM_HUMAN Afamin	69 kDa	7.63E+05	5.95E+05	0.78	0.5429
217	P11234-2	RALB	RALB_HUMAN Isoform 2 of Ras-related protein Ral-B	26 kDa	6.14E+06	4.01E+06	0.65	0.4285
218	P78386	KRT85	KRT85_HUMAN Keratin, type II cuticular Hb5	56 kDa	4.12E+05	6.72E+07	162.90	0.5010
219	Q5VTE0	EEF1A1P5	EF1A3_HUMAN Putative elongation factor 1-alpha-like 3	50 kDa	1.56E+06	1.06E+06	0.68	0.3214
220	Q14254	FLOT2	FLOT2_HUMAN Flotillin-2	47 kDa	4.31E+05	7.30E+05	1.69	0.3167
221	P12273	PIP	PIP_HUMAN Prolactin-inducible protein	17 kDa	1.92E+06	1.01E+06	0.53	0.0498
222	P00918	CA2	CAH2_HUMAN Carbonic anhydrase 2	29 kDa	4.63E+06	2.01E+06	0.43	0.2039
223	P06702	S100A9	S10A9_HUMAN Protein S100-A9	13 kDa	5.44E+06	7.34E+06	1.35	0.4125
224	P19013	KRT4	K2C4_HUMAN Keratin, type II cytoskeletal 4	57 kDa	7.25E+07	4.05E+07	0.56	0.2885
225	Q6Q788	APOA5	APOA5_HUMAN Apolipoprotein A-V	41 kDa	1.81E+06	5.11E+06	2.82	0.3208

SUPPLEMENTARY DATA

226	A0A0B4J1U7	IGHV6-1	HV601_HUMAN Immunoglobulin heavy variable 6-1	13 kDa	2.15E+07	2.26E+07	1.05	0.8826
227	P61160	ACTR2	ARP2_HUMAN Actin-related protein 2	45 kDa	4.45E+06	2.49E+06	0.56	0.2828
228	P06312	IGKV4-1	KV401_HUMAN Immunoglobulin kappa variable 4-1	13 kDa	6.66E+07	3.74E+07	0.56	0.3905
229	P0DOX2		IGA2_HUMAN Immunoglobulin alpha-2 heavy chain	49 kDa	4.26E+07	2.46E+07	0.58	0.2989
230	P00352	ALDH1A1	AL1A1_HUMAN Retinal dehydrogenase 1	55 kDa	9.71E+05	9.96E+05	1.02	0.9447
231	P00915	CA1	CAH1_HUMAN Carbonic anhydrase 1	29 kDa	9.83E+05	2.72E+06	2.77	0.0079
232	P09211	GSTP1	GSTP1_HUMAN Glutathione S-transferase P	23 kDa	6.92E+06	4.46E+06	0.64	0.3522
233	P35611-3	ADD1	ADDA_HUMAN Isoform 3 of Alpha-adducin	84 kDa	1.01E+06	1.00E+06	0.99	0.9828
234	P22392-2	NME2	NDKB_HUMAN Isoform 3 of Nucleoside diphosphate kinase B	30 kDa	4.72E+06	1.66E+06	0.35	0.1418
235	Q14344	GNA13	GNA13_HUMAN Guanine nucleotide-binding protein subunit alpha-13	44 kDa	8.61E+06	5.55E+06	0.64	0.1981
236	P12429	ANXA3	ANXA3_HUMAN Annexin A3	36 kDa	2.80E+06	5.09E+05	0.18	0.1296
237	P11169	SLC2A3	GTR3_HUMAN Solute carrier family 2, facilitated glucose transporter member 3	54 kDa	6.66E+06	3.68E+06	0.55	0.1429
238	P13611	VCAN	CSPG2_HUMAN Versican core protein	373 kDa	2.49E+05	3.65E+05	1.46	0.4150
239	Q08722-2	CD47	CD47_HUMAN Isoform OA3-293 of Leukocyte surface antigen CD47	32 kDa	1.26E+07	1.23E+07	0.97	0.9078
240	Q15907	RAB11B	RB11B_HUMAN Ras-related protein Rab-11B	24 kDa	5.36E+06	1.74E+06	0.33	0.1503
241	Q7Z794	KRT77	K2C1B_HUMAN Keratin, type II cytoskeletal 1b	62 kDa	1.72E+08	9.39E+07	0.55	0.2695
242	P22694-2	PRKACB	KAPCB_HUMAN Isoform 2 of cAMP-dependent protein kinase catalytic subunit beta	46 kDa	7.64E+05	4.45E+05	0.58	0.1253
243	Q01813	PFKP	PFKAP_HUMAN ATP-dependent 6-phosphofructokinase, platelet type	86 kDa	1.03E+06	1.31E+05	0.13	0.0679
244	O75558	STX11	STX11_HUMAN Syntaxin-11	33 kDa	3.69E+06	1.17E+06	0.32	0.0457
245	P60660-2	MYL6	MYL6_HUMAN Isoform Smooth muscle of Myosin light polypeptide 6	17 kDa	1.00E+07	5.49E+06	0.55	0.2350
246	Q13093	PLA2G7	PAFA_HUMAN Platelet-activating factor acetylhydrolase	50 kDa	1.00E+06	1.25E+06	1.25	0.6051
247	Q8WZ42-13	TTN	TITIN_HUMAN Isoform 13 of Titin	3831 kDa	#DIV/0!	6.77E+03	#DIV/0!	#DIV/0!
248	P02748	C9	CO9_HUMAN Complement component C9	63 kDa	5.76E+05	8.98E+05	1.56	0.1720
249	P31146	CORO1A	COR1A_HUMAN Coronin-1A	51 kDa	2.57E+06	1.42E+06	0.55	0.1704
250	P59998-3	ARPC4	ARPC4_HUMAN Isoform 3 of Actin-related protein 2/3 complex subunit 4	22 kDa	1.00E+07	3.39E+06	0.34	0.0626
251	P07203	GPX1	GPX1_HUMAN Glutathione peroxidase 1	22 kDa	5.53E+06	2.20E+06	0.40	0.1734
252	P22314-2	UBA1	UBA1_HUMAN Isoform 2 of Ubiquitin-like modifier-activating enzyme 1	114 kDa	1.50E+05	1.26E+05	0.84	0.6385
253	P26641-2	EEF1G	EF1G_HUMAN Isoform 2 of Elongation factor 1-gamma	56 kDa	9.78E+05	4.43E+05	0.45	0.1913
254	Q5T749	KPRP	KPRP_HUMAN Keratinocyte proline-rich protein	64 kDa	8.89E+05	1.94E+05	0.22	0.1294

© 2020. Muraoka S. et al. Published online at http://www.aginganddisease.org/EN/10.14336/AD.2020.0908

255	P05109	S100A8	S10A8_HUMAN Protein S100-A8	11 kDa	8.13E+06	5.97E+06	0.73	0.3944
256	P62879	GNB2	GBB2_HUMAN Guanine nucleotide-binding protein G(I)/G(S)/G(T) subunit beta-2	37 kDa	1.08E+07	6.01E+06	0.56	0.1185
257	P50502	ST13	F10A1_HUMAN Hsc70-interacting protein	41 kDa	1.57E+06	1.12E+06	0.71	0.4100
258	P07237	P4HB	PDIA1_HUMAN Protein disulfide-isomerase	57 kDa	1.61E+05	2.48E+05	1.54	0.3829
259	O14950	MYL12B	ML12B_HUMAN Myosin regulatory light chain 12B	20 kDa	2.36E+06	3.47E+06	1.47	0.5852
260	P48740	MASP1	MASP1_HUMAN Mannan-binding lectin serine protease 1	79 kDa	5.87E+06	7.86E+05	0.13	0.3670
261	P04083	ANXA1	ANXA1_HUMAN Annexin A1	39 kDa	6.96E+05	1.02E+06	1.47	0.6553
262	Q8IWA5-3	SLC44A2	CTL2_HUMAN Isoform 3 of Choline transporter-like protein 2	80 kDa	8.26E+05	6.54E+05	0.79	0.5599
263	P61225	RAP2B	RAP2B_HUMAN Ras-related protein Rap-2b	21 kDa	4.26E+06	2.35E+06	0.55	0.3226
264	Q9H4M9	EHD1	EHD1_HUMAN EH domain-containing protein 1	61 kDa	3.77E+06	1.63E+06	0.43	0.2164
265	P06331	IGHV4-34	HV434_HUMAN Immunoglobulin heavy variable 4-34	14 kDa	4.73E+06	7.09E+06	1.50	0.6420
266	P78371	CCT2	TCPB_HUMAN T-complex protein 1 subunit beta	57 kDa	4.15E+05	4.14E+05	1.00	0.9936
267	O00429-6	DNM1L	DNM1L_HUMAN Isoform 6 of Dynamin-1-like protein	83 kDa	9.01E+05	2.08E+05	0.23	0.2363
268	P40227	CCT6A	TCPZ_HUMAN T-complex protein 1 subunit zeta	58 kDa	6.06E+05	3.21E+05	0.53	0.4330
269	P69891	HBG1	HBG1_HUMAN Hemoglobin subunit gamma-1	16 kDa	6.33E+08	7.86E+08	1.24	0.6592
270	Q6KB66	KRT80	K2C80_HUMAN Keratin, type II cytoskeletal 80	51 kDa	7.04E+07	1.63E+07	0.23	0.2514
271	Q99497	PARK7	PARK7_HUMAN Protein DJ-1	20 kDa	3.81E+06	1.38E+06	0.36	0.2121
272	Q02094	RHAG	RHAG_HUMAN Ammonium transporter Rh type A	44 kDa	8.55E+06	8.93E+06	1.04	0.9083
273	Q15555-4	MAPRE2	MARE2_HUMAN Isoform 4 of Microtubule-associated protein RP/EB family member 2	31 kDa	4.85E+06	2.07E+06	0.43	0.2174
274	P11413-2	G6PD	G6PD_HUMAN Isoform Long of Glucose-6-phosphate 1-dehydrogenase	64 kDa	6.29E+05	4.13E+05	0.66	0.3967
275	P49913	CAMP	CAMP_HUMAN Cathelicidin antimicrobial peptide	19 kDa	6.11E+06	6.97E+06	1.14	0.7442
276	P62993	GRB2	GRB2_HUMAN Growth factor receptor-bound protein 2	25 kDa	1.78E+06	6.43E+05	0.36	0.2218
277	O95810	SDPR	SDPR_HUMAN Serum deprivation-response protein	47 kDa	7.60E+06	1.68E+06	0.22	0.0983
278	O15145	ARPC3	ARPC3_HUMAN Actin-related protein 2/3 complex subunit 3	21 kDa	2.27E+06	1.80E+06	0.79	0.6242
279	P05089-2	ARG1	ARGI1_HUMAN Isoform 2 of Arginase-1	36 kDa	2.25E+06	5.85E+05	0.26	0.0704
280	P81605	DCD	DCD_HUMAN Dermcidin	11 kDa	2.01E+07	1.05E+07	0.52	0.3274
281	P25789	PSMA4	PSA4_HUMAN Proteasome subunit alpha type-4	29 kDa	1.98E+06	9.85E+05	0.50	0.1284
282	Q01469	FABP5	FABP5_HUMAN Fatty acid-binding protein, epidermal	15 kDa	1.30E+07	2.05E+06	0.16	0.1940
283	Q8WWZ8	OIT3	OIT3_HUMAN Oncoprotein-induced transcript 3 protein	60 kDa	2.24E+06	2.74E+06	1.22	0.8476
284	P01624	IGKV3-15	KV315_HUMAN Immunoglobulin kappa variable 3-15	12 kDa	3.11E+07	3.88E+07	1.25	0.6370

© 2020. Muraoka S. et al. Published online at http://www.aginganddisease.org/EN/10.14336/AD.2020.0908

285	P02760	AMBP	AMBP_HUMAN Protein AMBP	39 kDa	9.28E+05	1.56E+06	1.68	0.0936
286	P08603	CFH	CFAH_HUMAN Complement factor H	139 kDa	2.58E+05	1.52E+05	0.59	0.3811
287	P50990	CCT8	TCPQ_HUMAN T-complex protein 1 subunit theta	60 kDa	4.11E+05	2.21E+05	0.54	0.1571
288	P52566	ARHGDIB	GDIR2_HUMAN Rho GDP-dissociation inhibitor 2	23 kDa	2.77E+06	1.75E+06	0.63	0.3537
289	Q9NRW1	RAB6B	RAB6B_HUMAN Ras-related protein Rab-6B	23 kDa	1.11E+07	5.85E+06	0.53	0.1624
290	P01833	PIGR	PIGR_HUMAN Polymeric immunoglobulin receptor	83 kDa	7.45E+05	4.20E+05	0.56	0.3758
291	Q9H4G4	GLIPR2	GAPR1_HUMAN Golgi-associated plant pathogenesis-related protein 1	17 kDa	3.12E+06	9.91E+05	0.32	0.0930
292	P13598	ICAM2	ICAM2_HUMAN Intercellular adhesion molecule 2	31 kDa	1.82E+06	1.05E+06	0.58	0.3630
293	P04792	HSPB1	HSPB1_HUMAN Heat shock protein beta-1	23 kDa	4.60E+06	2.93E+06	0.64	0.4007
294	P47755	CAPZA2	CAZA2_HUMAN F-actin-capping protein subunit alpha-2	33 kDa	3.66E+06	2.15E+06	0.59	0.2496
295	Q15762	CD226	CD226_HUMAN CD226 antigen	39 kDa	1.94E+06	1.29E+06	0.67	0.2765
296	P37837	TALDO1	TALDO_HUMAN Transaldolase	38 kDa	1.96E+06	3.26E+05	0.17	0.0761
297	Q9Y624	F11R	JAM1_HUMAN Junctional adhesion molecule A	33 kDa	4.02E+06	2.76E+06	0.69	0.2088
298	Q13790	APOF	APOF_HUMAN Apolipoprotein F	35 kDa	9.79E+06	1.50E+07	1.53	0.3062
299	P48426-2	PIP4K2A	PI42A_HUMAN Isoform 2 of Phosphatidylinositol 5-phosphate 4-kinase type-2 alpha	40 kDa	1.37E+06	5.41E+05	0.40	0.3778
300	P35612	ADD2	ADDB_HUMAN Beta-adducin	81 kDa	8.35E+05	1.32E+06	1.58	0.4867
301	O00161-2	SNAP23	SNP23_HUMAN Isoform SNAP-23b of Synaptosomal-associated protein 23	18 kDa	4.01E+06	1.62E+06	0.40	0.2722
302	P29350-3	PTPN6	PTN6_HUMAN Isoform 2 of Tyrosine-protein phosphatase non-receptor type 6	68 kDa	2.86E+05	2.49E+05	0.87	0.7929
303	Q13576	IQGAP2	IQGA2_HUMAN Ras GTPase-activating-like protein IQGAP2	181 kDa	2.13E+05	7.29E+04	0.34	0.3587
304	Q3ZCW2	LGALSL	LEGL_HUMAN Galectin-related protein	19 kDa	3.94E+06	2.23E+06	0.57	0.3462
305	Q96P63	SERPINB12	SPB12_HUMAN Serpin B12	46 kDa	3.07E+06	9.84E+05	0.32	0.2891
306	Q14019	COTL1	COTL1_HUMAN Coactosin-like protein	16 kDa	2.27E+06	9.92E+05	0.44	0.4135
307	P55058	PLTP	PLTP_HUMAN Phospholipid transfer protein	55 kDa	1.20E+06	1.05E+06	0.88	0.8424
308	O14818	PSMA7	PSA7_HUMAN Proteasome subunit alpha type-7	28 kDa	1.53E+06	1.04E+06	0.68	0.4095
309	O15511	ARPC5	ARPC5_HUMAN Actin-related protein 2/3 complex subunit 5	16 kDa	2.14E+06	3.16E+06	1.47	0.4602
310	P04745	AMY1A	AMY1_HUMAN Alpha-amylase 1	58 kDa	1.08E+07	1.76E+06	0.16	0.5532
311	Q08554-2	DSC1	DSC1_HUMAN Isoform 1B of Desmocollin-1	94 kDa	3.18E+06	3.34E+05	0.10	0.2337
312	P01700	IGLV1-47	LV147_HUMAN Immunoglobulin lambda variable 1-47	12 kDa	3.21E+07	1.35E+07	0.42	0.3336
313	P12110	COL6A2	CO6A2_HUMAN Collagen alpha-2(VI) chain	109 kDa	4.17E+05	7.13E+05	1.71	0.1740
314	P55209-2	NAP1L1	NP1L1_HUMAN Isoform 2 of Nucleosome assembly protein 1-like 1	43 kDa	1.58E+06	9.25E+05	0.59	0.2080
315	P04433	IGKV3-11	KV311_HUMAN Immunoglobulin kappa variable 3-11	13 kDa	4.56E+07	3.95E+07	0.87	0.8059

© 2020. Muraoka S. et al. Published online at http://www.aginganddisease.org/EN/10.14336/AD.2020.0908

316	P28289	TMOD1	TMOD1_HUMAN Tropomodulin-1	41 kDa	5.88E+05	1.12E+06	1.91	0.1664
317	P49368	CCT3	TCPG_HUMAN T-complex protein 1 subunit gamma	61 kDa	1.11E+06	7.25E+05	0.66	0.4192
318	P52565	ARHGDIA	GDIR1_HUMAN Rho GDP-dissociation inhibitor 1	23 kDa	3.09E+06	2.21E+06	0.72	0.4015
319	P53396-2	ACLY	ACLY_HUMAN Isoform 2 of ATP-citrate synthase	120 kDa	8.55E+04	1.06E+05	1.24	0.6925
320	P61626	LYZ	LYSC_HUMAN Lysozyme C	17 kDa	5.52E+06	4.59E+06	0.83	0.7515
321	Q8WWI5-2	SLC44A1	CTL1_HUMAN Isoform 2 of Choline transporter-like protein 1	73 kDa	4.86E+05	4.17E+05	0.86	0.6010
322	P29401-2	TKT	TKT_HUMAN Isoform 2 of Transketolase	69 kDa	4.61E+05	2.84E+05	0.62	0.2144
323	P40925-3	MDH1	MDHC_HUMAN Isoform 3 of Malate dehydrogenase, cytoplasmic	39 kDa	6.64E+05	4.46E+05	0.67	0.4410
324	P52907	CAPZA1	CAZA1_HUMAN F-actin-capping protein subunit alpha-1	33 kDa	5.00E+06	4.27E+06	0.86	0.7135
325	Q08495-2	DMTN	DEMA_HUMAN Isoform 2 of Dematin	43 kDa	1.45E+06	6.67E+05	0.46	0.3511
326	Q9UBW5	BIN2	BIN2_HUMAN Bridging integrator 2	62 kDa	3.72E+06	6.81E+05	0.18	0.1278
327	P62136	PPP1CA	PP1A_HUMAN Serine/threonine-protein phosphatase PP1-alpha catalytic subunit	38 kDa	8.20E+05	3.48E+05	0.42	0.2228
328	P01031	C5	CO5_HUMAN Complement C5	188 kDa	1.25E+05	5.39E+04	0.43	0.2822
329	P01701	IGLV1-51	LV151_HUMAN Immunoglobulin lambda variable 1-51	12 kDa	1.78E+07	1.15E+07	0.65	0.4085
330	P20851-2	C4BPB	C4BPB_HUMAN Isoform 2 of C4b-binding protein beta chain	28 kDa	3.26E+06	2.85E+06	0.88	0.8462
331	Q8N699	MYCT1	MYCT1_HUMAN Myc target protein 1	27 kDa	1.81E+06	1.26E+06	0.70	0.5775
332	P02745	C1QA	C1QA_HUMAN Complement C1q subcomponent subunit A	26 kDa	5.91E+06	5.63E+06	0.95	0.9013
333	P01861	IGHG4	IGHG4_HUMAN Immunoglobulin heavy constant gamma 4	36 kDa	9.39E+07	1.17E+08	1.25	0.2882
334	P0DP04	IGHV3-43D	HV43D_HUMAN Immunoglobulin heavy variable 3-43D	13 kDa	9.37E+07	4.13E+07	0.44	0.1468
335	P13987	CD59	CD59_HUMAN CD59 glycoprotein	14 kDa	6.82E+06	3.26E+06	0.48	0.0416
336	P16150	SPN	LEUK_HUMAN Leukosialin	40 kDa	1.43E+06	7.58E+05	0.53	0.2434
337	P01743	IGHV1-46	HV146_HUMAN Immunoglobulin heavy variable 1-46	13 kDa	2.71E+06	8.10E+06	2.99	0.1776
338	P51148-2	RAB5C	RAB5C_HUMAN Isoform 2 of Ras-related protein Rab-5C	27 kDa	1.68E+06	5.73E+05	0.34	0.0827
339	P01597	IGKV1-39	KV139_HUMAN Immunoglobulin kappa variable 1-39	13 kDa	2.69E+08	#DIV/0!	#DIV/0!	#DIV/0!
340	O75116	ROCK2	ROCK2_HUMAN Rho-associated protein kinase 2	161 kDa	2.06E+05	#DIV/0!	#DIV/0!	#DIV/0!
341	P60033	CD81	CD81_HUMAN CD81 antigen	26 kDa	2.89E+06	7.63E+06	2.65	0.0218
342	Q14974	KPNB1	IMB1_HUMAN Importin subunit beta-1	97 kDa	2.31E+05	1.99E+05	0.86	0.8158
343	O95866	G6B	G6B_HUMAN Protein G6b	26 kDa	5.52E+06	2.90E+06	0.53	0.3817
344	O00187	MASP2	MASP2_HUMAN Mannan-binding lectin serine protease 2	76 kDa	4.44E+06	2.62E+05	0.06	0.3802

© 2020. Muraoka S. et al. Published online at http://www.aginganddisease.org/EN/10.14336/AD.2020.0908

SUPPLEMENTARY DATA

345	P18054	ALOX12	LOX12_HUMAN Arachidonate 12-lipoxygenase, 12S-type	76 kDa	3.45E+05	1.51E+05	0.44	0.2982
346	P23284	PPIB	PPIB_HUMAN Peptidyl-prolyl cis-trans isomerase B	24 kDa	1.18E+06	5.80E+05	0.49	0.1536
347	Q07960	ARHGAP1	RHG01_HUMAN Rho GTPase-activating protein 1	50 kDa	6.28E+05	3.08E+05	0.49	0.3776
348	Q6UX06	OLFM4	OLFM4_HUMAN Olfactomedin-4	57 kDa	1.44E+05	3.51E+06	24.42	0.3233
349	P09871	C1S	C1S_HUMAN Complement C1s subcomponent	77 kDa	3.63E+05	3.27E+05	0.90	#DIV/0!
350	A0A0B4J1V0	IGHV3-15	HV315_HUMAN Immunoglobulin heavy variable 3-15	13 kDa	2.54E+07	2.17E+07	0.85	0.7587
351	O00592-2	PODXL	PODXL_HUMAN Isoform 2 of Podocalyxin	55 kDa	7.66E+05	1.06E+06	1.39	0.3457
352	Q06323	PSME1	PSME1_HUMAN Proteasome activator complex subunit 1	29 kDa	5.27E+05	3.39E+05	0.64	0.3179
353	P50991-2	CCT4	TCPD_HUMAN Isoform 2 of T-complex protein 1 subunit delta	55 kDa	1.88E+05	1.83E+05	0.97	0.9386
354	Q9Y6E0-2	STK24	STK24_HUMAN Isoform A of Serine/threonine-protein kinase 24	48 kDa	7.09E+05	3.00E+05	0.42	0.0986
355	A0A0A0MS15	IGHV3-49	HV349_HUMAN Immunoglobulin heavy variable 3-49	13 kDa	8.91E+06	6.66E+06	0.75	0.6031
356	P0DJI8	SAA1	SAA1_HUMAN Serum amyloid A-1 protein	14 kDa	7.47E+06	6.90E+06	0.92	0.8791
357	O60610-3	DIAPH1	DIAP1_HUMAN Isoform 3 of Protein diaphanous homolog 1	140 kDa	8.54E+04	8.28E+04	0.97	0.9375
358	Q5SQ64	LY6G6F	LY66F_HUMAN Lymphocyte antigen 6 complex locus protein G6f	32 kDa	3.32E+06	1.62E+06	0.49	0.1296
359	O75954	TSPAN9	TSN9_HUMAN Tetraspanin-9	27 kDa	2.89E+06	1.37E+06	0.48	0.1892
360	A0A0B4J1Y9	IGHV3-72	HV372_HUMAN Immunoglobulin heavy variable 3-72	13 kDa	3.45E+07	3.73E+07	1.08	0.8862
361	A0A0C4DH68	IGKV2-24	KV224_HUMAN Immunoglobulin kappa variable 2-24	13 kDa	1.24E+07	2.50E+07	2.02	0.2377
362	P05154	SERPINA5	IPSP_HUMAN Plasma serine protease inhibitor	46 kDa	9.71E+06	3.64E+05	0.04	0.4930
363	P30044-2	PRDX5	PRDX5_HUMAN Isoform Cytoplasmic+peroxisomal of Peroxiredoxin-5, mitochondrial	17 kDa	3.34E+06	1.34E+06	0.40	0.2286
364	Q6UWD8	C16orf54	CP054_HUMAN Transmembrane protein C16orf54	24 kDa	1.67E+06	1.12E+06	0.67	0.5002
365	P35613-2	BSG	BASI_HUMAN Isoform 2 of Basigin	29 kDa	9.34E+05	7.05E+05	0.75	#DIV/0!
366	O00151	PDLIM1	PDLI1_HUMAN PDZ and LIM domain protein 1	36 kDa	2.92E+06	8.47E+05	0.29	0.2241
367	O76074-2	PDE5A	PDE5A_HUMAN Isoform PDE5A2 of cGMP-specific 3',5'-cyclic phosphodiesterase	95 kDa	6.36E+05	1.21E+05	0.19	0.2178
368	Q08431-3	MFGE8	MFGM_HUMAN Isoform 3 of Lactadherin	38 kDa	4.40E+05	1.35E+06	3.06	0.1133
369	Q8NG11	TSPAN14	TSN14_HUMAN Tetraspanin-14	31 kDa	1.57E+06	1.23E+06	0.78	0.4848
370	Q86VP6-2	CAND1	CAND1_HUMAN Isoform 2 of Cullin-associated NEDD8-dissociated protein 1	118 kDa	2.53E+05	3.86E+05	1.52	#DIV/0!
371	P00492	HPRT1	HPRT_HUMAN Hypoxanthine-guanine phosphoribosyltransferase	25 kDa	2.73E+05	7.51E+05	2.75	0.1219
372	Q95IE3	HLA-DRB1	2B1C_HUMAN HLA class II histocompatibility antigen, DRB1-12 beta chain	30 kDa	#DIV/0!	4.66E+05	#DIV/0!	#DIV/0!

© 2020. Muraoka S. et al. Published online at http://www.aginganddisease.org/EN/10.14336/AD.2020.0908

373	P20618	PSMB1	PSB1_HUMAN Proteasome subunit beta type-1	26 kDa	6.26E+05	5.55E+05	0.89	0.8406
374	Q684P5-2	RAP1GAP2	RPGP2_HUMAN Isoform 2 of Rap1 GTPase-activating protein 2	78 kDa	4.44E+05	1.31E+05	0.30	0.2106
375	Q99808-2	SLC29A1	S29A1_HUMAN Isoform 2 of Equilibrative nucleoside transporter 1	59 kDa	4.61E+05	5.89E+05	1.28	0.5817
376	P27701	CD82	CD82_HUMAN CD82 antigen	30 kDa	6.47E+05	8.68E+05	1.34	0.2738
377	P05771-2	PRKCB	KPCB_HUMAN Isoform Beta-II of Protein kinase C beta type	77 kDa	4.35E+05	1.08E+05	0.25	0.1863
378	P13796	LCP1	PLSL_HUMAN Plastin-2	70 kDa	#DIV/0!	3.52E+05	#DIV/0!	#DIV/0!
379	P09525	ANXA4	ANXA4_HUMAN Annexin A4	36 kDa	5.04E+05	3.19E+05	0.63	0.4877
380	P01599	IGKV1-17	KV117_HUMAN Immunoglobulin kappa variable 1-17	13 kDa	6.88E+06	4.08E+06	0.59	0.3025
381	P19971	TYMP	TYPH_HUMAN Thymidine phosphorylase	50 kDa	5.58E+05	3.32E+05	0.59	0.4873
382	P27797	CALR	CALR_HUMAN Calreticulin	48 kDa	4.91E+05	3.30E+05	0.67	0.6076
383	P30101	PDIA3	PDIA3_HUMAN Protein disulfide-isomerase A3	57 kDa	2.76E+05	4.50E+05	1.63	0.5397
384	P36871	PGM1	PGM1_HUMAN Phosphoglucomutase-1	61 kDa	3.53E+05	1.15E+05	0.33	0.0660
385	Q99439	CNN2	CNN2_HUMAN Calponin-2	34 kDa	2.31E+06	1.09E+06	0.47	0.1783
386	Q6IBS0	TWF2	TWF2_HUMAN Twinfilin-2	40 kDa	1.53E+06	6.38E+05	0.42	0.2155
387	P02724-2	GYPA	GLPA_HUMAN Isoform 2 of Glycophorin-A	14 kDa	5.20E+07	4.08E+07	0.79	0.6330
388	Q9P126-2	CLEC1B	CLC1B_HUMAN Isoform 2 of C-type lectin domain family 1 member B	23 kDa	2.38E+06	8.16E+05	0.34	0.0899
389	P23526	AHCY	SAHH_HUMAN Adenosylhomocysteinase	48 kDa	3.46E+05	6.08E+05	1.76	0.4680
390	Q9Y6W5	WASF2	WASF2_HUMAN Wiskott-Aldrich syndrome protein family member 2	54 kDa	1.07E+06	3.69E+05	0.35	0.0711
391	Q7Z7G0	ABI3BP	TARSH_HUMAN Target of Nesh-SH3	119 kDa	1.23E+05	3.81E+05	3.10	#DIV/0!
392	P47929	LGALS7	LEG7_HUMAN Galectin-7	15 kDa	8.85E+06	5.33E+05	0.06	0.3316
393	P08697-2	SERPINF2	A2AP_HUMAN Isoform 2 of Alpha-2-antiplasmin	48 kDa	6.61E+05	3.52E+05	0.53	0.5215
394	P55160	NCKAP1L	NCKPL_HUMAN Nck-associated protein 1-like	128 kDa	4.63E+05	3.07E+05	0.66	0.3144
395	P16070-11	CD44	CD44_HUMAN Isoform 11 of CD44 antigen	47 kDa	6.80E+05	7.85E+05	1.16	0.7315
396	P05107	ITGB2	ITB2_HUMAN Integrin beta-2	85 kDa	2.23E+05	3.65E+05	1.64	#DIV/0!
397	Q15942	ZYX	ZYX_HUMAN Zyxin	61 kDa	2.48E+06	1.30E+06	0.52	#DIV/0!
398	P05023-4	ATP1A1	AT1A1_HUMAN Isoform 4 of Sodium/potassium-transporting ATPase subunit alpha-1	113 kDa	2.22E+05	1.11E+05	0.50	0.1968
399	O15117-3	FYB	FYB_HUMAN Isoform 3 of FYN-binding protein	92 kDa	6.91E+05	2.86E+05	0.41	0.1328
400	A0A075B6P5	IGKV2-28	KV228_HUMAN Immunoglobulin kappa variable 2-28	13 kDa	3.06E+07	1.27E+08	4.16	0.1180
401	Q08188	TGM3	TGM3_HUMAN Protein-glutamine gamma-glutamyltransferase E	77 kDa	5.96E+05	7.93E+05	1.33	0.8301
402	Q96PD5-2	PGLYRP2	PGRP2_HUMAN Isoform 2 of N-acetylmuramoyl-L-alanine amidase	68 kDa	2.68E+05	4.88E+05	1.82	0.2482
403	P62328	TMSB4X	TYB4_HUMAN Thymosin beta-4	5 kDa	1.39E+07	1.20E+07	0.86	0.7753

© 2020. Muraoka S. et al. Published online at http://www.aginganddisease.org/EN/10.14336/AD.2020.0908

404	P61769	B2M	B2MG_HUMAN Beta-2-microglobulin	14 kDa	2.27E+06	3.30E+06	1.45	0.4609
405	P62158	CALM1	CALM_HUMAN Calmodulin	17 kDa	5.51E+06	3.41E+06	0.62	0.5281
406	O75131	CPNE3	CPNE3_HUMAN Copine-3	60 kDa	1.76E+05	6.34E+05	3.60	#DIV/0!
407	Q16851	UGP2	UGPA_HUMAN UTP--glucose-1-phosphate uridylyltransferase	57 kDa	1.26E+06	2.55E+05	0.20	0.1967
408	P48643	CCT5	TCPE_HUMAN T-complex protein 1 subunit epsilon	60 kDa	1.63E+05	1.65E+05	1.01	0.9836
409	O75874	IDH1	IDHC_HUMAN Isocitrate dehydrogenase [NADP] cytoplasmic	47 kDa	4.65E+05	9.90E+04	0.21	0.1122
410	P18669	PGAM1	PGAM1_HUMAN Phosphoglycerate mutase 1	29 kDa	1.13E+06	7.44E+05	0.66	0.4918
411	O00186	STXBP3	STXB3_HUMAN Syntaxin-binding protein 3	68 kDa	1.75E+05	1.83E+05	1.05	0.8695
412	P03952	KLKB1	KLKB1_HUMAN Plasma kallikrein	71 kDa	1.12E+07	1.22E+05	0.01	#DIV/0!
413	P35237	SERPINB6	SPB6_HUMAN Serpin B6	43 kDa	1.33E+06	2.89E+05	0.22	0.1848
414	Q13835-2	PKP1	PKP1_HUMAN Isoform 1 of Plakophilin-1	80 kDa	3.18E+05	#DIV/0!	#DIV/0!	#DIV/0!
415	P62805	HIST1H4A	H4_HUMAN Histone H4	11 kDa	2.63E+06	1.81E+06	0.69	0.3195
416	Q8IZP0-12	ABI1	ABI1_HUMAN Isoform 12 of Abl interactor 1	54 kDa	2.57E+06	2.58E+06	1.00	0.9981
417	P02452	COL1A1	CO1A1_HUMAN Collagen alpha-1(I) chain	139 kDa	1.91E+06	2.31E+05	0.12	#DIV/0!
418	Q02818	NUCB1	NUCB1_HUMAN Nucleobindin-1	54 kDa	2.02E+05	1.60E+05	0.79	0.7616
419	P04921-2	GYPC	GLPC_HUMAN Isoform Glycophorin-D of Glycophorin-C	11 kDa	9.57E+06	8.05E+06	0.84	0.5647
420	Q9H299	SH3BGRL3	SH3L3_HUMAN SH3 domain-binding glutamic acid-rich-like protein 3	10 kDa	1.36E+07	1.08E+07	0.79	0.6614
421	O75695	RP2	XRP2_HUMAN Protein XRP2	40 kDa	4.20E+05	3.14E+05	0.75	0.4724
422	P42768	WAS	WASP_HUMAN Wiskott-Aldrich syndrome protein	53 kDa	8.21E+05	2.60E+05	0.32	0.3924
423	P08133-2	ANXA6	ANXA6_HUMAN Isoform 2 of Annexin A6	72 kDa	1.77E+05	4.84E+04	0.27	0.2090
424	P16109	SELP	LYAM3_HUMAN P-selectin	91 kDa	1.32E+06	9.60E+04	0.07	0.2153
425	P31944	CASP14	CASPE_HUMAN Caspase-14	28 kDa	1.06E+06	1.02E+06	0.96	0.9659
426	Q13867	BLMH	BLMH_HUMAN Bleomycin hydrolase	53 kDa	4.99E+05	5.42E+05	1.09	0.7448
427	Q99829	CPNE1	CPNE1_HUMAN Copine-1	59 kDa	4.64E+05	9.42E+05	2.03	0.3583
428	Q9NR31	SAR1A	SAR1A_HUMAN GTP-binding protein SAR1a	22 kDa	3.11E+06	8.55E+05	0.27	0.2101
429	P60900	PSMA6	PSA6_HUMAN Proteasome subunit alpha type-6	27 kDa	9.00E+05	7.19E+05	0.80	0.5933
430	Q96KP4	CNDP2	CNDP2_HUMAN Cytosolic non-specific dipeptidase	53 kDa	3.33E+05	1.12E+05	0.34	0.2718
431	Q15746-11	MYLK	MYLK_HUMAN Isoform 9 of Myosin light chain kinase, smooth muscle	110 kDa	4.24E+05	1.69E+05	0.40	0.2449
432	P17987	TCP1	TCPA_HUMAN T-complex protein 1 subunit alpha	60 kDa	2.82E+05	1.88E+05	0.66	0.2159
433	P14151	SELL	LYAM1_HUMAN L-selectin	42 kDa	3.24E+05	2.61E+05	0.81	0.5190
434	P01040	CSTA	CYTA_HUMAN Cystatin-A	11 kDa	3.55E+06	1.21E+06	0.34	0.4575
435	P62826	RAN	RAN_HUMAN GTP-binding nuclear protein Ran	24 kDa	1.14E+06	6.10E+05	0.53	0.2512
436	Q15848	ADIPOQ	ADIPO_HUMAN Adiponectin	26 kDa	1.65E+07	1.75E+06	0.11	0.3816
437	Q96C24	SYTL4	SYTL4_HUMAN Synaptotagmin-like protein 4	76 kDa	3.37E+05	1.50E+05	0.44	0.0857

© 2020. Muraoka S. et al. Published online at http://www.aginganddisease.org/EN/10.14336/AD.2020.0908

438	Q8N392-2	ARHGAP 18	RHG18_HUMAN Isoform 2 of Rho GTPase-activating protein 18	70 kDa	2.37E+05	1.33E+05	0.56	0.2460
439	P80748	IGLV3-21	LV321_HUMAN Immunoglobulin lambda variable 3-21	12 kDa	5.53E+06	4.55E+06	0.82	0.7601
440	P10599	TXN	THIO_HUMAN Thioredoxin	12 kDa	8.29E+06	5.30E+06	0.64	0.5031
441	Q5D862	FLG2	FILA2_HUMAN Filaggrin-2	248 kDa	2.55E+05	3.56E+05	1.39	0.7468
442	Q6YHK3	CD109	CD109_HUMAN CD109 antigen	162 kDa	6.00E+04	5.33E+04	0.89	0.8303
443	O75368	SH3BGRL	SH3L1_HUMAN SH3 domain-binding glutamic acid-rich-like protein	13 kDa	1.82E+06	6.43E+05	0.35	0.2559
444	P09486	SPARC	SPRC_HUMAN SPARC	35 kDa	1.27E+06	1.19E+06	0.94	0.8647
445	P61019	RAB2A	RAB2A_HUMAN Ras-related protein Rab-2A	24 kDa	5.97E+05	1.28E+05	0.21	0.1840
446	P49407-2	ARRB1	ARRB1_HUMAN Isoform 1B of Beta-arrestin-1	46 kDa	6.00E+05	2.55E+05	0.43	0.2254
447	P08174-7	CD55	DAF_HUMAN Isoform 7 of Complement decay-accelerating factor	59 kDa	2.41E+05	2.33E+05	0.97	0.9305
448	P31948-2	STIP1	STIP1_HUMAN Isoform 2 of Stress-induced-phosphoprotein 1	68 kDa	4.83E+05	1.01E+05	0.21	0.2159
449	P25787	PSMA2	PSA2_HUMAN Proteasome subunit alpha type-2	26 kDa	3.35E+05	2.45E+05	0.73	0.3460
450	P01903	HLA-DRA	DRA_HUMAN HLA class II histocompatibility antigen, DR alpha chain	29 kDa	#DIV/0!	1.19E+06	#DIV/0!	#DIV/0!
451	P25705-2	ATP5A1	ATPA_HUMAN Isoform 2 of ATP synthase subunit alpha, mitochondrial	54 kDa	#DIV/0!	1.16E+06	#DIV/0!	#DIV/0!
452	P36955	SERPINF1	PEDF_HUMAN Pigment epithelium-derived factor	46 kDa	4.96E+05	5.93E+05	1.20	0.7998
453	Q96RW7-2	HMCN1	HMCN1_HUMAN Isoform 2 of Hemicentin-1	600 kDa	3.50E+04	1.43E+05	4.08	#DIV/0!
454	P18577-9	RHCE	RHCE_HUMAN Isoform 4g of Blood group Rh(CE) polypeptide	27 kDa	3.96E+06	4.00E+06	1.01	0.9857
455	P30626-2	SRI	SORCN_HUMAN Isoform 2 of Sorcin	20 kDa	7.61E+05	1.24E+06	1.63	0.4975
456	P28066	PSMA5	PSA5_HUMAN Proteasome subunit alpha type-5	26 kDa	1.64E+06	9.69E+05	0.59	0.1050
457	P29622	SERPINA 4	KAIN_HUMAN Kallistatin	49 kDa	2.44E+05	3.38E+05	1.38	0.5129
458	Q14642	INPP5A	I5P1_HUMAN Type I inositol 1,4,5-trisphosphate 5-phosphatase	48 kDa	3.87E+05	1.92E+05	0.50	0.2757
459	Q13642-5	FHL1	FHL1_HUMAN Isoform 5 of Four and a half LIM domains protein 1	34 kDa	1.10E+06	4.30E+06	3.90	0.2082
460	Q06033-2	ITIH3	ITIH3_HUMAN Isoform 2 of Inter-alpha-trypsin inhibitor heavy chain H3	99 kDa	3.43E+04	1.08E+05	3.16	0.0138
461	Q16555-2	DPYSL2	DPYL2_HUMAN Isoform 2 of Dihydropyrimidinase-related protein 2	58 kDa	6.90E+05	3.17E+05	0.46	#DIV/0!
462	O75563	SKAP2	SKAP2_HUMAN Src kinase-associated phosphoprotein 2	41 kDa	2.27E+06	4.31E+05	0.19	0.2363
463	P08473	MME	NEP_HUMAN Neprilysin	86 kDa	3.81E+04	7.63E+05	20.00	#DIV/0!
464	P27487	DPP4	DPP4_HUMAN Dipeptidyl peptidase 4	88 kDa	9.93E+04	1.60E+06	16.08	#DIV/0!
465	P42357	HAL	HUTH_HUMAN Histidine ammonia-lyase	73 kDa	2.20E+06	6.56E+05	0.30	#DIV/0!
466	P25788-2	PSMA3	PSA3_HUMAN Isoform 2 of Proteasome subunit alpha type-3	28 kDa	7.93E+05	5.11E+05	0.64	0.5694

© 2020. Muraoka S. et al. Published online at http://www.aginganddisease.org/EN/10.14336/AD.2020.0908

SUPPLEMENTARY DATA

467	Q15758	SLC1A5	AAAT_HUMAN Neutral amino acid transporter B(0)	57 kDa	5.91E+05	3.72E+05	0.63	0.3620
468	Q9BR76	CORO1B	COR1B_HUMAN Coronin-1B	54 kDa	8.70E+05	3.45E+05	0.40	0.2966
469	Q9NZT1	CALML5	CALL5_HUMAN Calmodulin-like protein 5	16 kDa	4.02E+06	1.30E+06	0.32	0.1913
470	P49721	PSMB2	PSB2_HUMAN Proteasome subunit beta type-2	23 kDa	5.23E+05	5.71E+05	1.09	0.7891
471	P42892-2	ECE1	ECE1_HUMAN Isoform A of Endothelin-converting enzyme 1	86 kDa	4.32E+05	2.21E+04	0.05	#DIV/0!
472	Q99685	MGLL	MGLL_HUMAN Monoglyceride lipase	33 kDa	1.42E+05	2.56E+05	1.80	0.2762
473	P01137	TGFB1	TGFB1_HUMAN Transforming growth factor beta-1	44 kDa	2.02E+05	1.97E+05	0.97	0.9363
474	P30153	PPP2R1A	2AAA_HUMAN Serine/threonine-protein phosphatase 2A 65 kDa regulatory subunit A alpha isoform	65 kDa	2.89E+05	#DIV/0!	#DIV/0!	#DIV/0!
475	P19440	GGT1	GGT1_HUMAN Gamma-glutamyltranspeptidase 1	61 kDa	7.03E+05	1.89E+06	2.70	#DIV/0!
476	P23634-3	ATP2B4	AT2B4_HUMAN Isoform ZA of Plasma membrane calcium-transporting ATPase 4	128 kDa	#DIV/0!	1.70E+05	#DIV/0!	#DIV/0!
477	O15031	PLXNB2	PLXB2_HUMAN Plexin-B2	205 kDa	9.77E+04	4.60E+03	0.05	#DIV/0!
478	P02788-2	LTF	TRFL_HUMAN Isoform DeltaLf of Lactotransferrin	73 kDa	6.30E+05	1.10E+05	0.17	0.2646
479	Q9UGM3-2	DMBT1	DMBT1_HUMAN Isoform 2 of Deleted in malignant brain tumors 1 protein	194 kDa	5.64E+05	9.88E+06	17.50	#DIV/0!
480	P04430	IGKV1-16	KV116_HUMAN Immunoglobulin kappa variable 1-16	13 kDa	1.03E+07	7.68E+06	0.75	0.7474
481	P30566	ADSL	PUR8_HUMAN Adenylosuccinate lyase	55 kDa	6.39E+04	5.82E+04	0.91	0.7721
482	Q15485	FCN2	FCN2_HUMAN Ficolin-2	34 kDa	1.47E+06	6.18E+05	0.42	0.6472
483	O00139-5	KIF2A	KIF2A_HUMAN Isoform 5 of Kinesin-like protein KIF2A	78 kDa	1.24E+05	1.04E+05	0.84	0.4046
484	Q9BX67	JAM3	JAM3_HUMAN Junctional adhesion molecule C	35 kDa	1.20E+06	2.20E+05	0.18	0.2986
485	P13861-2	PRKAR2A	KAP2_HUMAN Isoform 2 of cAMP-dependent protein kinase type II-alpha regulatory subunit	43 kDa	1.90E+06	3.83E+06	2.02	0.2926
486	Q9NVA2-2	11-Sep	SEP11_HUMAN Isoform 2 of Septin-11	51 kDa	1.54E+06	6.50E+05	0.42	0.5358
487	P01116-2	KRAS	RASK_HUMAN Isoform 2B of GTPase KRas	21 kDa	#DIV/0!	1.79E+06	#DIV/0!	#DIV/0!
488	O43639	NCK2	NCK2_HUMAN Cytoplasmic protein NCK2	43 kDa	#DIV/0!	4.59E+05	#DIV/0!	#DIV/0!
489	Q96A08	HIST1H2BA	H2B1A_HUMAN Histone H2B type 1-A	14 kDa	#DIV/0!	4.32E+05	#DIV/0!	#DIV/0!
490	O43488	AKR7A2	ARK72_HUMAN Aflatoxin B1 aldehyde reductase member 2	40 kDa	2.36E+05	#DIV/0!	#DIV/0!	#DIV/0!
491	P14625	HSP90B1	ENPL_HUMAN Endoplasmin	92 kDa	3.22E+05	1.50E+05	0.46	0.2557
492	P11226	MBL2	MBL2_HUMAN Mannose-binding protein C	26 kDa	3.87E+05	6.23E+05	1.61	0.5574
493	P60981	DSTN	DEST_HUMAN Destrin	19 kDa	2.47E+06	9.48E+05	0.38	0.2914
494	P22234	PAICS	PUR6_HUMAN Multifunctional protein ADE2	47 kDa	8.88E+04	1.12E+05	1.26	0.6965
495	P31939-2	ATIC	PUR9_HUMAN Isoform 2 of Bifunctional purine biosynthesis protein PURH	65 kDa	3.08E+05	7.22E+04	0.23	0.5218
496	P22748	CA4	CAH4_HUMAN Carbonic anhydrase 4	35 kDa	1.27E+05	2.79E+05	2.20	0.0995

SUPPLEMENTARY DATA

497	Q7L9L4-2	MOB1B	MOB1B_HUMAN Isoform 2 of MOB kinase activator 1B	25 kDa	5.54E+05	5.72E+05	1.03	0.9426
498	Q9NX76	CMTM6	CKLF6_HUMAN CKLF-like MARVEL transmembrane domain-containing protein 6	20 kDa	4.91E+06	3.50E+05	0.07	0.2653
499	P01780	IGHV3-7	HV307_HUMAN Immunoglobulin heavy variable 3-7	13 kDa	7.04E+07	7.03E+07	1.00	0.9982
500	Q9HCN6-2	GP6	GPVI_HUMAN Isoform 2 of Platelet glycoprotein VI	35 kDa	1.31E+06	8.67E+05	0.66	#DIV/0!
501	Q9H939	PSTPIP2	PPIP2_HUMAN Proline-serine-threonine phosphatase-interacting protein 2	39 kDa	2.48E+06	6.52E+05	0.26	#DIV/0!
502	P11021	HSPA5	GRP78_HUMAN 78 kDa glucose-regulated protein	72 kDa	3.45E+05	2.33E+05	0.67	0.7214
503	Q92619-2	HMHA1	HMHA1_HUMAN Isoform 2 of Minor histocompatibility protein HA-1	126 kDa	3.34E+05	7.63E+04	0.23	0.2274
504	P05543	SERPINA7	THBG_HUMAN Thyroxine-binding globulin	46 kDa	1.19E+05	1.78E+05	1.49	0.3089
505	P16152	CBR1	CBR1_HUMAN Carbonyl reductase [NADPH] 1	30 kDa	8.11E+05	8.50E+04	0.10	0.3052
506	Q01433-2	AMPD2	AMPD2_HUMAN Isoform Ex1A-2-3 of AMP deaminase 2	92 kDa	4.02E+05	6.87E+04	0.17	0.2993
507	P09543-2	CNP	CN37_HUMAN Isoform CNPI of 2',3'-cyclic-nucleotide 3'-phosphodiesterase	45 kDa	2.96E+05	1.99E+05	0.67	#DIV/0!
508	Q96RT1-5	ERBIN	ERBIN_HUMAN Isoform 5 of Erbin	152 kDa	6.96E+04	4.84E+04	0.70	0.6677
509	A0A0C4DH24	IGKV6-21	KV621_HUMAN Immunoglobulin kappa variable 6-21	12 kDa	1.09E+06	1.71E+06	1.56	0.5971
510	P37235	HPCAL1	HPCL1_HUMAN Hippocalcin-like protein 1	22 kDa	#DIV/0!	2.20E+05	#DIV/0!	#DIV/0!
511	A0A075B6K4	IGLV3-10	LV310_HUMAN Immunoglobulin lambda variable 3-10	12 kDa	1.06E+07	1.25E+07	1.17	0.9070
512	Q15019-3	2-Sep	SEPT2_HUMAN Isoform 3 of Septin-2	43 kDa	5.51E+05	3.05E+05	0.55	0.3594
513	Q9UBQ6	EXTL2	EXTL2_HUMAN Exostosin-like 2	37 kDa	6.06E+07	#DIV/0!	#DIV/0!	#DIV/0!
514	P23083	IGHV1-2	HV102_HUMAN Immunoglobulin heavy variable 1-2	13 kDa	3.43E+06	6.25E+06	1.82	0.3260
515	Q06187-2	BTK	BTK_HUMAN Isoform BTK-C of Tyrosine-protein kinase BTK	80 kDa	4.93E+05	8.09E+04	0.16	#DIV/0!
516	P62330	ARF6	ARF6_HUMAN ADP-ribosylation factor 6	20 kDa	1.52E+06	9.14E+05	0.60	0.2996
517	P16930	FAH	FAAA_HUMAN Fumarylacetoacetase	46 kDa	2.06E+05	3.12E+05	1.51	0.7235
518	P10809	HSPD1	CH60_HUMAN 60 kDa heat shock protein, mitochondrial	61 kDa	2.01E+06	2.42E+05	0.12	0.5370
519	P20701-2	ITGAL	ITAL_HUMAN Isoform 2 of Integrin alpha-L	134 kDa	2.80E+04	6.61E+04	2.36	#DIV/0!
520	Q9Y6M1-1	IGF2BP2	IF2B2_HUMAN Isoform 2 of Insulin-like growth factor 2 mRNA-binding protein 2	62 kDa	1.93E+05	7.28E+05	3.76	#DIV/0!
521	P55786	NPEPPS	PSA_HUMAN Puromycin-sensitive aminopeptidase	103 kDa	9.78E+04	8.50E+04	0.87	#DIV/0!
522	Q9NP81-2	SARS2	SYSM_HUMAN Isoform 2 of Serine--tRNA ligase, mitochondrial	58 kDa	7.52E+05	1.74E+06	2.32	#DIV/0!
523	B2RUZ4	SMIM1	SMIM1_HUMAN Small integral membrane protein 1	9 kDa	5.56E+06	2.76E+06	0.50	0.4744

© 2020. Muraoka S. et al. Published online at http://www.aginganddisease.org/EN/10.14336/AD.2020.0908

524	Q93084-2	ATP2A3	AT2A3_HUMAN Isoform SERCA3A of Sarcoplasmic/endoplasmic reticulum calcium ATPase 3	109 kDa	2.46E+05	3.23E+05	1.32	#DIV/0!
525	Q96QA5	GSDMA	GSDMA_HUMAN Gasdermin-A	49 kDa	6.21E+05	1.70E+05	0.27	0.2183
526	P16112-2	ACAN	PGCA_HUMAN Isoform 2 of Aggrecan core protein	246 kDa	#DIV/0!	1.35E+05	#DIV/0!	#DIV/0!
527	P07741	APRT	APT_HUMAN Adenine phosphoribosyltransferase	20 kDa	1.43E+06	5.36E+05	0.38	0.1523
528	P51452	DUSP3	DUS3_HUMAN Dual specificity protein phosphatase 3	20 kDa	1.62E+06	4.41E+05	0.27	0.1857
529	Q5JSH3-3	WDR44	WDR44_HUMAN Isoform 3 of WD repeat-containing protein 44	49 kDa	2.03E+05	6.94E+04	0.34	0.0810
530	Q15691	MAPRE1	MARE1_HUMAN Microtubule-associated protein RP/EB family member 1	30 kDa	1.97E+06	1.70E+05	0.09	0.2358
531	Q27J81-2	INF2	INF2_HUMAN Isoform 2 of Inverted formin-2	135 kDa	1.83E+05	7.48E+04	0.41	0.4086
532	O60234	GMFG	GMFG_HUMAN Glia maturation factor gamma	17 kDa	6.51E+05	4.83E+05	0.74	#DIV/0!
533	A0A0B4J1V2	IGHV2-26	HV226_HUMAN Immunoglobulin heavy variable 2-26	13 kDa	1.06E+06	1.01E+06	0.95	0.9500
534	P13164	IFITM1	IFM1_HUMAN Interferon-induced transmembrane protein 1	14 kDa	5.68E+06	3.47E+06	0.61	0.5213
535	P68402	PAFAH1B2	PA1B2_HUMAN Platelet-activating factor acetylhydrolase IB subunit beta	26 kDa	4.35E+05	4.89E+05	1.12	0.8773
536	P15090	FABP4	FABP4_HUMAN Fatty acid-binding protein, adipocyte	15 kDa	#DIV/0!	7.31E+05	#DIV/0!	#DIV/0!
537	Q16181	7-Sep	SEPT7_HUMAN Septin-7	51 kDa	1.11E+06	7.74E+05	0.70	#DIV/0!
538	P02792	FTL	FRIL_HUMAN Ferritin light chain	20 kDa	#DIV/0!	2.95E+07	#DIV/0!	#DIV/0!
539	Q8TDL5	BPIFB1	BPIB1_HUMAN BPI fold-containing family B member 1	52 kDa	5.63E+05	#DIV/0!	#DIV/0!	#DIV/0!
540	P07358	C8B	CO8B_HUMAN Complement component C8 beta chain	67 kDa	1.73E+05	1.92E+05	1.11	#DIV/0!
541	P21291	CSRP1	CSRP1_HUMAN Cysteine and glycine-rich protein 1	21 kDa	3.24E+06	7.56E+05	0.23	0.4568
542	Q9HDC9	APMAP	APMAP_HUMAN Adipocyte plasma membrane-associated protein	46 kDa	1.97E+05	2.91E+05	1.48	0.6030
543	P02753	RBP4	RET4_HUMAN Retinol-binding protein 4	23 kDa	2.29E+06	7.42E+05	0.32	0.0565
544	P05156	CFI	CFAI_HUMAN Complement factor I	66 kDa	2.14E+05	1.15E+05	0.54	0.4422
545	Q5T750	XP32	XP32_HUMAN Skin-specific protein 32	26 kDa	3.44E+06	4.42E+05	0.13	0.2266
546	A0A075B6I0	IGLV8-61	LV861_HUMAN Immunoglobulin lambda variable 8-61	13 kDa	3.73E+07	2.82E+07	0.76	0.7427
547	Q9Y376	CAB39	CAB39_HUMAN Calcium-binding protein 39	40 kDa	1.53E+05	1.65E+05	1.08	0.8344
548	P60842	EIF4A1	IF4A1_HUMAN Eukaryotic initiation factor 4A-I	46 kDa	3.20E+06	#DIV/0!	#DIV/0!	#DIV/0!
549	Q05682-3	CALD1	CALD1_HUMAN Isoform 3 of Caldesmon	64 kDa	#DIV/0!	5.65E+05	#DIV/0!	#DIV/0!
550	P06576	ATP5B	ATPB_HUMAN ATP synthase subunit beta, mitochondrial	57 kDa	2.13E+05	2.32E+05	1.09	#DIV/0!
551	P29508-2	SERPINB3	SPB3_HUMAN Isoform 2 of Serpin B3	39 kDa	3.95E+05	3.39E+05	0.86	#DIV/0!

552	Q8IZ83	ALDH16A1	A16A1_HUMAN Aldehyde dehydrogenase family 16 member A1	85 kDa	3.68E+04	2.04E+05	5.56	#DIV/0!
553	P12821	ACE	ACE_HUMAN Angiotensin-converting enzyme	150 kDa	#DIV/0!	7.75E+04	#DIV/0!	#DIV/0!
554	P27824-2	CANX	CALX_HUMAN Isoform 2 of Calnexin	72 kDa	1.85E+05	2.66E+05	1.43	0.7188
555	P04179	SOD2	SODM_HUMAN Superoxide dismutase [Mn], mitochondrial	25 kDa	7.21E+05	9.51E+05	1.32	#DIV/0!
556	Q8TF42	UBASH3B	UBS3B_HUMAN Ubiquitin-associated and SH3 domain-containing protein B	73 kDa	2.56E+05	4.87E+04	0.19	#DIV/0!
557	O95858	TSPAN15	TSN15_HUMAN Tetraspanin-15	33 kDa	3.96E+05	6.94E+05	1.75	0.3662
558	P14324-2	FDPS	FPPS_HUMAN Isoform 2 of Farnesyl pyrophosphate synthase	41 kDa	1.10E+06	1.86E+05	0.17	#DIV/0!
559	Q15517	CDSN	CDSN_HUMAN Corneodesmosin	52 kDa	1.45E+06	1.64E+05	0.11	#DIV/0!
560	P09972	ALDOC	ALDOC_HUMAN Fructose-bisphosphate aldolase C	39 kDa	#DIV/0!	3.25E+05	#DIV/0!	#DIV/0!
561	Q6ZVX7	NCCRP1	FBX50_HUMAN F-box only protein 50	31 kDa	4.14E+05	2.05E+05	0.49	0.4758
562	Q9UIB8-2	CD84	SLAF5_HUMAN Isoform 2 of SLAM family member 5	38 kDa	1.42E+06	4.02E+05	0.28	#DIV/0!
563	A0A0C4DH67	IGKV1-8	KV108_HUMAN Immunoglobulin kappa variable 1-8	13 kDa	5.46E+07	1.62E+07	0.30	0.5871
564	P28074-3	PSMB5	PSB5_HUMAN Isoform 3 of Proteasome subunit beta type-5	18 kDa	7.84E+05	3.96E+05	0.50	0.2887
565	Q96FJ2	DYNLL2	DYL2_HUMAN Dynein light chain 2, cytoplasmic	10 kDa	1.89E+06	2.83E+05	0.15	#DIV/0!
566	Q9H2K8	TAOK3	TAOK3_HUMAN Serine/threonine-protein kinase TAO3	105 kDa	#DIV/0!	2.18E+05	#DIV/0!	#DIV/0!
567	Q16539-4	MAPK14	MK14_HUMAN Isoform Exip of Mitogen-activated protein kinase 14	35 kDa	1.72E+06	#DIV/0!	#DIV/0!	#DIV/0!
568	O94919	ENDOD1	ENDD1_HUMAN Endonuclease domain-containing 1 protein	55 kDa	4.50E+05	1.92E+05	0.43	#DIV/0!
569	P00748	F12	FA12_HUMAN Coagulation factor XII	68 kDa	7.66E+06	#DIV/0!	#DIV/0!	#DIV/0!
570	Q9NQ84-2	GPRC5C	GPC5C_HUMAN Isoform 2 of G-protein coupled receptor family C group 5 member C	49 kDa	1.14E+06	2.00E+06	1.75	#DIV/0!
571	Q8WXI7	MUC16	MUC16_HUMAN Mucin-16	1519 kDa	#DIV/0!	3.08E+04	#DIV/0!	#DIV/0!
572	P22735	TGM1	TGM1_HUMAN Protein-glutamine gamma-glutamyltransferase K	90 kDa	#DIV/0!	8.34E+04	#DIV/0!	#DIV/0!
573	P07360	C8G	CO8G_HUMAN Complement component C8 gamma chain	22 kDa	3.64E+05	1.39E+05	0.38	0.0723
574	A0A0J9YX35	IGHV3-64D	HV64D_HUMAN Immunoglobulin heavy variable 3-64D	13 kDa	1.40E+07	1.53E+06	0.11	0.1036
575	P10301	RRAS	RRAS_HUMAN Ras-related protein R-Ras	23 kDa	1.09E+06	3.93E+05	0.36	0.4207
576	P48735-2	IDH2	IDHP_HUMAN Isoform 2 of Isocitrate dehydrogenase [NADP], mitochondrial	45 kDa	4.68E+05	1.11E+05	0.24	0.2593
577	P54709	ATP1B3	AT1B3_HUMAN Sodium/potassium-transporting ATPase subunit beta-3	32 kDa	1.02E+06	3.71E+05	0.37	#DIV/0!
578	Q9BUL8	PDCD10	PDC10_HUMAN Programmed cell death protein 10	25 kDa	7.41E+05	7.75E+05	1.05	0.9525

© 2020. Muraoka S. et al. Published online at http://www.aginganddisease.org/EN/10.14336/AD.2020.0908

SUPPLEMENTARY DATA

579	Q09666	AHNAK	AHNK_HUMAN Neuroblast differentiation-associated protein AHNAK	629 kDa	3.66E+04	1.10E+04	0.30	#DIV/0!
580	Q9NRY6	PLSCR3	PLS3_HUMAN Phospholipid scramblase 3	32 kDa	6.78E+05	4.23E+05	0.62	0.3729
581	O14745	SLC9A3R1	NHRF1_HUMAN Na(+)/H(+) exchange regulatory cofactor NHE-RF1	39 kDa	#DIV/0!	2.20E+05	#DIV/0!	#DIV/0!
582	P28062	PSMB8	PSB8_HUMAN Proteasome subunit beta type-8	30 kDa	2.17E+05	5.97E+04	0.28	0.2591
583	P84095	RHOG	RHOG_HUMAN Rho-related GTP-binding protein RhoG	21 kDa	3.06E+06	3.70E+05	0.12	#DIV/0!
584	Q13404-8	UBE2V1	UB2V1_HUMAN Isoform 6 of Ubiquitin-conjugating enzyme E2 variant 1	12 kDa	1.11E+06	6.82E+05	0.62	#DIV/0!
585	Q15382	RHEB	RHEB_HUMAN GTP-binding protein Rheb	20 kDa	8.85E+05	1.70E+05	0.19	0.3424
586	P41250	GARS	GARS_HUMAN Glycine--tRNA ligase	83 kDa	7.68E+04	3.86E+04	0.50	#DIV/0!
587	P54578-2	USP14	UBP14_HUMAN Isoform 2 of Ubiquitin carboxyl-terminal hydrolase 14	52 kDa	8.61E+04	1.01E+05	1.17	0.7272
588	P01766	IGHV3-13	HV313_HUMAN Immunoglobulin heavy variable 3-13	13 kDa	1.65E+08	7.15E+07	0.43	0.1189
589	Q5TZA2	CROCC	CROCC_HUMAN Rootletin	229 kDa	2.38E+05	#DIV/0!	#DIV/0!	#DIV/0!
590	P41240	CSK	CSK_HUMAN Tyrosine-protein kinase CSK	51 kDa	#DIV/0!	2.15E+05	#DIV/0!	#DIV/0!
591	Q8TF66-2	LRRC15	LRC15_HUMAN Isoform 2 of Leucine-rich repeat-containing protein 15	65 kDa	#DIV/0!	2.74E+06	#DIV/0!	#DIV/0!
592	P61163	ACTR1A	ACTZ_HUMAN Alpha-centractin	43 kDa	2.63E+05	4.52E+05	1.72	#DIV/0!
593	Q9UQB8	BAIAP2	BAIP2_HUMAN Brain-specific angiogenesis inhibitor 1-associated protein 2	61 kDa	#DIV/0!	1.64E+05	#DIV/0!	#DIV/0!
594	Q9BWP8-9	COLEC11	COL11_HUMAN Isoform 9 of Collectin-11	29 kDa	#DIV/0!	6.02E+05	#DIV/0!	#DIV/0!
595	P08123	COL1A2	CO1A2_HUMAN Collagen alpha-2(I) chain	129 kDa	#DIV/0!	7.90E+04	#DIV/0!	#DIV/0!
596	P0DOX3		IGD_HUMAN Immunoglobulin delta heavy chain	56 kDa	2.59E+05	5.39E+05	2.08	0.0082
597	P42229-2	STAT5A	STA5A_HUMAN Isoform 2 of Signal transducer and activator of transcription 5A	87 kDa	1.71E+05	7.12E+04	0.42	0.5503
598	B0I1T2	MYO1G	MYO1G_HUMAN Unconventional myosin-Ig	116 kDa	3.96E+04	6.12E+04	1.55	#DIV/0!
599	P00451	F8	FA8_HUMAN Coagulation factor VIII	267 kDa	#DIV/0!	5.25E+04	#DIV/0!	#DIV/0!
600	Q02156	PRKCE	KPCE_HUMAN Protein kinase C epsilon type	84 kDa	#DIV/0!	9.08E+05	#DIV/0!	#DIV/0!
601	A0A0B4J1Y8	IGLV9-49	LV949_HUMAN Immunoglobulin lambda variable 9-49	13 kDa	1.20E+08	3.74E+05	0.00	#DIV/0!
602	Q9Y315	DERA	DEOC_HUMAN Deoxyribose-phosphate aldolase	35 kDa	1.11E+05	2.31E+05	2.07	#DIV/0!
603	P49747-2	COMP	COMP_HUMAN Isoform 2 of Cartilage oligomeric matrix protein	77 kDa	#DIV/0!	7.37E+05	#DIV/0!	#DIV/0!
604	A0A075B6S5	IGKV1-27	KV127_HUMAN Immunoglobulin kappa variable 1-27	13 kDa	4.21E+07	1.28E+07	0.31	0.2392
605	P09488	GSTM1	GSTM1_HUMAN Glutathione S-transferase Mu 1	26 kDa	7.76E+04	6.11E+05	7.87	#DIV/0!
606	A0A0B4J1V6	IGHV3-73	HV373_HUMAN Immunoglobulin heavy variable 3-73	13 kDa	3.77E+07	4.65E+07	1.23	0.7658

© 2020. Muraoka S. et al. Published online at http://www.aginganddisease.org/EN/10.14336/AD.2020.0908

607	P49908	SELENOP	SEPP1_HUMAN Selenoprotein P	43 kDa	7.13E+04	2.39E+05	3.35	#DIV/0!
608	Q86YZ3	HRNR	HORN_HUMAN Hornerin	282 kDa	2.03E+05	3.37E+05	1.66	0.7326
609	P98172	EFNB1	EFNB1_HUMAN Ephrin-B1	38 kDa	2.56E+05	1.49E+05	0.58	0.5224
610	P02750	LRG1	A2GL_HUMAN Leucine-rich alpha-2-glycoprotein	38 kDa	8.13E+04	1.79E+05	2.20	#DIV/0!
611	P24557-2	TBXAS1	THAS_HUMAN Isoform 2 of Thromboxane-A synthase	53 kDa	1.29E+05	2.34E+05	1.81	#DIV/0!
612	O75915	ARL6IP5	PRAF3_HUMAN PRA1 family protein 3	22 kDa	7.66E+05	1.27E+06	1.65	#DIV/0!
613	Q9NQW7-3	XPNPEP1	XPP1_HUMAN Isoform 3 of Xaa-Pro aminopeptidase 1	75 kDa	2.46E+05	#DIV/0!	#DIV/0!	#DIV/0!
614	P49591	SARS	SYSC_HUMAN Serine--tRNA ligase, cytoplasmic	59 kDa	5.54E+05	1.04E+05	0.19	#DIV/0!
615	Q15582	TGFBI	BGH3_HUMAN Transforming growth factor-beta-induced protein ig-h3	75 kDa	#DIV/0!	8.29E+04	#DIV/0!	#DIV/0!
616	Q12974-4	PTP4A2	TP4A2_HUMAN Isoform 4 of Protein tyrosine phosphatase type IVA 2	16 kDa	1.08E+06	1.09E+05	0.10	#DIV/0!
617	Q5THJ4-2	VPS13D	VP13D_HUMAN Isoform 2 of Vacuolar protein sorting-associated protein 13D	489 kDa	#DIV/0!	1.72E+04	#DIV/0!	#DIV/0!
618	Q8IW75	SERPINA12	SPA12_HUMAN Serpin A12	47 kDa	2.78E+05	#DIV/0!	#DIV/0!	#DIV/0!
619	Q9NQC3-3	RTN4	RTN4_HUMAN Isoform 3 of Reticulon-4	22 kDa	1.38E+06	#DIV/0!	#DIV/0!	#DIV/0!
620	Q9Y6C2	EMILIN1	EMIL1_HUMAN EMILIN-1	107 kDa	2.16E+05	2.48E+05	1.15	#DIV/0!
621	P23141-2	CES1	EST1_HUMAN Isoform 2 of Liver carboxylesterase 1	63 kDa	4.04E+05	1.49E+05	0.37	#DIV/0!
622	Q9Y277-2	VDAC3	VDAC3_HUMAN Isoform 2 of Voltage-dependent anion-selective channel protein 3	31 kDa	7.33E+05	4.70E+05	0.64	#DIV/0!
623	P05141	SLC25A5	ADT2_HUMAN ADP/ATP translocase 2	33 kDa	6.59E+04	3.52E+05	5.34	#DIV/0!
624	P00568	AK1	KAD1_HUMAN Adenylate kinase isoenzyme 1	22 kDa	1.27E+06	3.37E+05	0.26	#DIV/0!
625	P02794	FTH1	FRIH_HUMAN Ferritin heavy chain	21 kDa	1.81E+05	7.52E+05	4.16	#DIV/0!
626	P05166	PCCB	PCCB_HUMAN Propionyl-CoA carboxylase beta chain, mitochondrial	58 kDa	5.61E+06	5.83E+05	0.10	#DIV/0!
627	P05362	ICAM1	ICAM1_HUMAN Intercellular adhesion molecule 1	58 kDa	8.10E+04	2.19E+05	2.70	0.3655
628	P11597-2	CETP	CETP_HUMAN Isoform 2 of Cholesteryl ester transfer protein	48 kDa	#DIV/0!	2.66E+05	#DIV/0!	#DIV/0!
629	Q13085-4	ACACA	ACACA_HUMAN Isoform 4 of Acetyl-CoA carboxylase 1	270 kDa	2.54E+04	1.80E+05	7.10	#DIV/0!
630	Q9UI42-2	CPA4	CBPA4_HUMAN Isoform 2 of Carboxypeptidase A4	44 kDa	2.83E+05	#DIV/0!	#DIV/0!	#DIV/0!
631	Q14697-2	GANAB	GANAB_HUMAN Isoform 2 of Neutral alpha-glucosidase AB	109 kDa	7.40E+04	2.12E+04	0.29	#DIV/0!
632	A8K2U0	A2ML1	A2ML1_HUMAN Alpha-2-macroglobulin-like protein 1	161 kDa	8.63E+04	1.09E+05	1.27	#DIV/0!
633	P54577	YARS	SYYC_HUMAN Tyrosine--tRNA ligase, cytoplasmic	59 kDa	5.45E+05	5.14E+04	0.09	#DIV/0!
634	P08571	CD14	CD14_HUMAN Monocyte differentiation antigen CD14	40 kDa	#DIV/0!	1.22E+05	#DIV/0!	#DIV/0!
635	P28070	PSMB4	PSB4_HUMAN Proteasome subunit beta type-4	29 kDa	5.01E+05	4.38E+05	0.87	#DIV/0!

© 2020. Muraoka S. et al. Published online at http://www.aginganddisease.org/EN/10.14336/AD.2020.0908

636	P28482	MAPK1	MK01_HUMAN Mitogen-activated protein kinase 1	41 kDa	1.72E+05	1.15E+05	0.67	0.3007
637	Q9NZQ3-3	NCKIPSD	SPN90_HUMAN Isoform 3 of NCK-interacting protein with SH3 domain	78 kDa	8.88E+04	1.05E+05	1.18	0.7625
638	P0C7H8	KRTAP2-3	KRA23_HUMAN Keratin-associated protein 2-3	13 kDa	#DIV/0!	1.88E+07	#DIV/0!	#DIV/0!
639	O76013	KRT36	KRT36_HUMAN Keratin, type I cuticular Ha6	52 kDa	#DIV/0!	1.96E+07	#DIV/0!	#DIV/0!
640	O00602	FCN1	FCN1_HUMAN Ficolin-1	35 kDa	1.07E+06	3.79E+05	0.36	#DIV/0!
641	P13671	C6	CO6_HUMAN Complement component C6	105 kDa	#DIV/0!	7.45E+04	#DIV/0!	#DIV/0!
642	P25786-2	PSMA1	PSA1_HUMAN Isoform Long of Proteasome subunit alpha type-1	30 kDa	6.72E+05	7.16E+05	1.07	#DIV/0!
643	P14174	MIF	MIF_HUMAN Macrophage migration inhibitory factor	12 kDa	1.03E+06	1.50E+06	1.46	#DIV/0!
644	O60496	DOK2	DOK2_HUMAN Docking protein 2	45 kDa	2.95E+05	7.55E+04	0.26	#DIV/0!
645	Q9BXJ4-2	C1QTNF3	C1QT3_HUMAN Isoform 2 of Complement C1q tumor necrosis factor-related protein 3	22 kDa	1.01E+07	1.44E+06	0.14	#DIV/0!
646	P0DMM9-2	SULT1A3	ST1A3_HUMAN Isoform 2 of Sulfotransferase 1A3	22 kDa	1.13E+06	2.73E+05	0.24	#DIV/0!
647	P13501	CCL5	CCL5_HUMAN C-C motif chemokine 5	10 kDa	4.67E+06	6.71E+05	0.14	#DIV/0!
648	A0A0C4DH31	IGHV1-18	HV118_HUMAN Immunoglobulin heavy variable 1-18	13 kDa	7.34E+06	4.13E+06	0.56	0.2111
649	A0A0B4J1X8	IGHV3-43	HV343_HUMAN Immunoglobulin heavy variable 3-43	13 kDa	1.01E+08	3.98E+07	0.39	#DIV/0!
650	Q9BYR8	KRTAP3-1	KRA31_HUMAN Keratin-associated protein 3-1	11 kDa	#DIV/0!	2.12E+07	#DIV/0!	#DIV/0!
651	O14556	GAPDHS	G3PT_HUMAN Glyceraldehyde-3-phosphate dehydrogenase, testis-specific	45 kDa	#DIV/0!	3.47E+06	#DIV/0!	#DIV/0!
652	P33176	KIF5B	KINH_HUMAN Kinesin-1 heavy chain	110 kDa	9.74E+04	#DIV/0!	#DIV/0!	#DIV/0!
653	Q8IUC0	KRTAP13-1	KR131_HUMAN Keratin-associated protein 13-1	18 kDa	#DIV/0!	7.78E+06	#DIV/0!	#DIV/0!
654	P02741	CRP	CRP_HUMAN C-reactive protein	25 kDa	2.42E+06	#DIV/0!	#DIV/0!	#DIV/0!
655	O95497	VNN1	VNN1_HUMAN Pantetheinase	57 kDa	#DIV/0!	5.57E+05	#DIV/0!	#DIV/0!
656	Q9NP55-2	BPIFA1	BPIA1_HUMAN Isoform 2 of BPI fold-containing family A member 1	25 kDa	#DIV/0!	1.56E+06	#DIV/0!	#DIV/0!
657	A0A087WSX0	IGLV5-45	LV545_HUMAN Immunoglobulin lambda variable 5-45	13 kDa	#DIV/0!	4.03E+06	#DIV/0!	#DIV/0!
658	Q12929	EPS8	EPS8_HUMAN Epidermal growth factor receptor kinase substrate 8	92 kDa	#DIV/0!	1.34E+05	#DIV/0!	#DIV/0!
659	P10619-2	CTSA	PPGB_HUMAN Isoform 2 of Lysosomal protective protein	52 kDa	8.22E+04	1.18E+05	1.44	#DIV/0!
660	Q9BSJ8-2	ESYT1	ESYT1_HUMAN Isoform 2 of Extended synaptotagmin-1	124 kDa	#DIV/0!	2.57E+04	#DIV/0!	#DIV/0!
661	Q9HAB8	PPCS	PPCS_HUMAN Phosphopantothenate--cysteine ligase	34 kDa	#DIV/0!	6.43E+04	#DIV/0!	#DIV/0!
662	Q9UBG3	CRNN	CRNN_HUMAN Cornulin	54 kDa	4.84E+04	2.08E+05	4.30	#DIV/0!
663	O15551	CLDN3	CLD3_HUMAN Claudin-3	23 kDa	2.40E+06	#DIV/0!	#DIV/0!	#DIV/0!

664	A0A0G2JS06	IGLV5-39	LV539_HUMAN Immunoglobulin lambda variable 5-39	13 kDa	1.06E+06	5.87E+06	5.55	#DIV/0!
665	P31323	PRKAR2B	KAP3_HUMAN cAMP-dependent protein kinase type II-beta regulatory subunit	46 kDa	7.72E+05	1.47E+05	0.19	#DIV/0!
666	Q08AD1-2	CAMSAP2	CAMP2_HUMAN Isoform 2 of Calmodulin-regulated spectrin-associated protein 2	165 kDa	1.30E+05	#DIV/0!	#DIV/0!	#DIV/0!
667	P04080	CSTB	CYTB_HUMAN Cystatin-B	11 kDa	#DIV/0!	2.03E+06	#DIV/0!	#DIV/0!
668	P23280-3	CA6	CAH6_HUMAN Isoform 3 of Carbonic anhydrase 6	29 kDa	1.23E+06	#DIV/0!	#DIV/0!	#DIV/0!
669	P50895	BCAM	BCAM_HUMAN Basal cell adhesion molecule	67 kDa	#DIV/0!	2.88E+05	#DIV/0!	#DIV/0!
670	Q9BYQ5	KRTAP4-6	KRA46_HUMAN Keratin-associated protein 4-6	22 kDa	#DIV/0!	9.58E+06	#DIV/0!	#DIV/0!
671	Q9ULL4-2	PLXNB3	PLXB3_HUMAN Isoform 2 of Plexin-B3	209 kDa	5.54E+04	#DIV/0!	#DIV/0!	#DIV/0!
672	P35443	THBS4	TSP4_HUMAN Thrombospondin-4	106 kDa	#DIV/0!	3.12E+05	#DIV/0!	#DIV/0!
673	A0A0C4DH43	IGHV2-70D	HV70D_HUMAN Immunoglobulin heavy variable 2-70D	13 kDa	6.76E+06	#DIV/0!	#DIV/0!	#DIV/0!
674	P32241-2	VIPR1	VIPR1_HUMAN Isoform Long of Vasoactive intestinal polypeptide receptor 1	55 kDa	9.88E+05	8.69E+05	0.88	0.6948
675	Q6A163	KRT39	K1C39_HUMAN Keratin, type I cytoskeletal 39	56 kDa	#DIV/0!	5.07E+04	#DIV/0!	#DIV/0!

[a]The value shows iBAQ intensity by Scaffold software. [b]The statistical significance of the differences were calculated using Student's t.test.

Supplementary Table 3. Identification of the EV and non-EV marker proteins.

Category	Gene name	Uniprot ID
	Transmembrane or GPI anchored proteins associated to plasma membrane and endosomes<br>For all EVs	
1a	CD81	P60033
	CD82	P27701
	CD47	Q08722
	GNAI2	P04899
	GNAQ	P50148
	GNAZ	P19086
	GNA13	Q14344
	HLA-A	P04439
	HLA-B	P01889
	ITGA2B	P08514
	ITGA6	P23229
	ITGA2	P17301
	ITGAL	P20701
	ITGB3	P05106
	ITGB1	P05556
	ITGB2	P05107
	EMMPRIN (BSG)	P35613

© 2020. Muraoka S. et al. Published online at http://www.aginganddisease.org/EN/10.14336/AD.2020.0908

	ADAM10	O14672
	CD55	P08174
	CD59	P13987
1b	CD9	P21926
	PECAM1	P16284
	CD45	P08575
	CD41 (ITGA2B)	P08514
	CD42a (GP9)	P14770
	GYPA	P02724
	CD14	P08571
Cytosolic proteins recovered in EVs<br>For all EVs		
2a	ALIX (PDCD6IP)	Q8WUM4
	FLOT1	O75955
	FLOT2	Q14254
	EHD3	Q9NZN3
	RHOA	P61586
	ANXA2	P07355
	ANXA5	P08758
	ANXA7	P20073
	ANXA11	P50995
	ANXA3	P12429
	ANXA1	P04083
	ANXA4	P09525
	ANXA6	P08133
	HSPA8	P11142
	ARF6	P62330
2b	HSP70 (HSPA1A)	P0DMV8
	ACTC1	P68032
	TUBB1	Q9H4B7
	TUBA1A	Q71U36
	GAPDH	P04406
Major components of non-EV co-isolated structures<br>For non-EV (contaminant)		
3a	APOE	P02649
	APOA1	P02647
	APOA2	P02652
	APOB100	P04114
	ALB	P02768
Analysis of protein of this category is required<br>when claiming specific analysis of small EVs		

© 2020. Muraoka S. et al. Published online at http://www.aginganddisease.org/EN/10.14336/AD.2020.0908

	EVs (large oncosomes, large EVs)	
	For small EVs	
4c (ER, Golgi)	calnexin (CANX)	P27824
	Grp94 (HSP90B1)	P14625
	BIP (HSPA5)	P11021
4d (autophagosome, other)	ACTN1	P12814
	Analysis of protein of this category is required when claiming functional activities	
	functional activites	
	For functional component	
5a (Cytokines)	TGFB1	P01137
5b (adhesion)	FN1	P02751
	COL6A3	P12111
	COL6A1	P12109
	COL6A2	P12110
	COL1A1	P02452
	COL1A2	P08123
	MFGE8	Q08431
	LGAL3BP	Q08380
	CD5L	O43866
	fetuin-A (AHSG)	P02765
Tetraspanin	TSPAN9	O75954
	TSPAN14	Q8NG11
	TSPAN15	O95858
ESCRT machinery	Rab5c	P51148
	Rab11b	Q15907
	Rab7a	P51149
	Rab27b	O00194
	coronin1b	Q9BR76
	syntenin-1(SDCBP)	O00560

Supplementary Table 4. An area under the ROC curve (AUC) for combination of tau or p-tau181 and differential expressed proteins.

Gene name	single	t-tau	p-tau$_{181}$
t-tau	0.736		
p-tau$_{181}$	0.715	0.736	0.736
RELN	0.846	0.846	0.846
CA1	0.849	0.944	0.926

© 2020. Muraoka S. et al. Published online at http://www.aginganddisease.org/EN/10.14336/AD.2020.0908

COL6A3	0.740	0.500	0.500
COL6A1	0.870	0.870	0.870
STX11	0.747	0.956	0.944
APOA4	0.714	0.819	0.714
SVEP1	0.833	0.833	0.833
VWF	0.774	0.736	0.774
SERPIND1	0.738	0.736	0.500
HSPA8	0.876	0.932	0.909
CD59	0.773	0.736	0.773
HSPA1A	0.778	0.778	0.778
TPM3	0.766	0.886	0.871
GP1BB	0.774	0.854	0.833
HLAA	0.744	0.875	0.774
RAB10	0.809	0.809	0.809
LPA	0.768	0.768	0.768

References

[1] Thery C, Witwer KW, Aikawa E, Alcaraz MJ, Anderson JD, Andriantsitohaina R, et al (2018). Minimal information for studies of extracellular vesicles 2018 (MISEV2018): a position statement of the International Society for Extracellular Vesicles and update of the MISEV2014 guidelines. J Extracell Vesicles, 7:1535750.

[2] Zhang Z, Song M, Liu X, Kang SS, Kwon I-S, Duong DM, et al (2014). Cleavage of tau by asparagine endopeptidase mediates the neurofibrillary pathology in Alzheimer's disease. Nat Med, 20:1254–62. AD-2020-0401R1

[3] Kurbatskaya K, Phillips EC, Croft CL, Dentoni G, Hughes MM, Wade MA, et al (2016). Upregulation of calpain activity precedes tau phosphorylation and loss of synaptic proteins in Alzheimer's disease brain. Acta Neuropathol Commun, 4:34.

[4] Muraoka S, DeLeo AM, Sethi MK, Yukawa-Takamatsu K, Yang Z, Ko J, et al (2020). Proteomic and biological profiling of extracellular vesicles from Alzheimer's disease human brain tissues. Alzheimers Dement, 16:896–907.

[5] Ko J, Baldassano SN, Loh P-L, Kording K, Litt B, Issadore D (2018). Machine learning to detect signatures of disease in liquid biopsies - a user's guide. Lab Chip, 18:395–405.

[6] Ahn S-M, Byun K, Cho K, Kim JY, Yoo JS, Kim D, et al (2008). Human microglial cells synthesize albumin in brain. PLoS ONE, 3:e2829.

[7] Stern RA, Adler CH, Chen K, Navitsky M, Luo J, Dodick DW, et al (2019). Tau Positron- Emission Tomography in Former National Football League Players. N Engl J Med, 380:1716–25.

[8] Nemani SK, Notari S, Cali I, Alvarez VE, Kofskey D, Cohen M, et al (2018). Cooccurrence of chronic traumatic encephalopathy and prion disease. Acta Neuropathol Commun, 6:140–14.

[9] Adams JW, Alvarez VE, Mez J, Huber BR, Tripodis Y, Xia W, et al (2018). Lewy Body Pathology and Chronic Traumatic Encephalopathy Associated With Contact Sports. J Neuropathol Exp Neurol, 77:757–68.

© 2020. Muraoka S. et al. Published online at http://www.aginganddisease.org/EN/10.14336/AD.2020.0908
